# ChemR23 activation reprograms macrophages toward a less inflammatory phenotype and dampens carcinoma progression

**DOI:** 10.3389/fimmu.2023.1196731

**Published:** 2023-07-19

**Authors:** Margot Lavy, Vanessa Gauttier, Alison Dumont, Florian Chocteau, Sophie Deshayes, Judith Fresquet, Virginie Dehame, Isabelle Girault, Charlène Trilleaud, Stéphanie Neyton, Caroline Mary, Philippe Juin, Nicolas Poirier, Sophie Barillé-Nion, Christophe Blanquart

**Affiliations:** ^1^ OSE Immunotherapeutics, Nantes, France; ^2^ Nantes Université, Inserm UMR 1307, CNRS UMR 6075, Université d’Angers, CRCI2NA, Nantes, France; ^3^ Nantes Université, CHU Nantes, service de pneumologie, l'institut du thorax, Nantes, France; ^4^ ICO René Gauducheau, Saint Herblain, France

**Keywords:** macrophage, cancer, ChemR23 receptor, agonist, antibody

## Abstract

**Introduction:**

Tumor Associated Macrophages (TAM) are a major component of the tumor environment and their accumulation often correlates with poor prognosis by contributing to local inflammation, inhibition of anti-tumor immune response and resistance to anticancer treatments. In this study, we thus investigated the anti-cancer therapeutic interest to target ChemR23, a receptor of the resolution of inflammation expressed by macrophages, using an agonist monoclonal antibody, αChemR23.

**Methods:**

Human GM-CSF, M-CSF and Tumor Associated Macrophage (TAM)-like macrophages were obtained by incubation of monocytes from healthy donors with GM-CSF, M-CSF or tumor cell supernatants (Breast cancer (BC) or malignant pleural mesothelioma (MPM) cells). The effects of αChemR23 on macrophages were studied at the transcriptomic, protein and functional level. Datasets from The Cancer Genome Atlas (TCGA) were used to study *CMKLR1* expression, coding for ChemR23, in BC and MPM tumors. *In vivo*, αChemR23 was evaluated on overall survival, metastasis development and transcriptomic modification of the metastatic niche using a model of resected triple negative breast cancer.

**Results:**

We show that ChemR23 is expressed at higher levels in M-CSF and tumor cell supernatant differentiated macrophages (TAM-like) than in GM-CSF-differentiated macrophages. ChemR23 activation triggered by αChemR23 deeply modulates M-CSF and TAM-like macrophages including profile of cell surface markers, cytokine secretion, gene mRNA expression and immune functions. The expression of ChemR23 coding gene (*CMKLR1*) strongly correlates to TAM markers in human BC tumors and MPM and its histological detection in these tumors mainly corresponds to TAM expression. *In vivo*, treatment with αChemR23 agonist increased mouse survival and decreased metastasis occurrence in a model of triple-negative BC in correlation with modulation of TAM phenotype in the metastatic niche.

**Conclusion:**

These results open an attractive opportunity to target TAM and the resolution of inflammation pathways through ChemR23 to circumvent TAM pro-tumoral effects.

## Introduction

Inflammation is a hallmark of cancer and cancer cells can hijack inflammatory mechanisms to promote their own growth and survival (1). Activation of pro-tumorigenic factors and secretion of pro-inflammatory cytokines by tumor cells and/or immune cells present in the tumor microenvironment (TME), including macrophages, contribute to tumor promotion (2). This inflammatory TME favors all stages of tumor progression such as angiogenesis and metastases and often modulates responses to cancer treatment (3). Inflammation is a natural and physiological process triggered after injury or infection that aims to restore tissue homeostasis and normally resolved spontaneously in a few days. However, when improperly terminated, inflammation evolves towards a chronic form contributing to tissue damages. Chronic inflammation is mainly associated with abnormal non-phlogistic clearance of apoptotic cells (efferocytosis) by macrophages and a defect of the resolution of inflammation (4). Anti-inflammatory drugs were shown to limit tumor inflammation however, in parallel they dampen both innate and adaptive immune responses, so their use in oncology was overall disappointing. The resolution of inflammation is an active immunological process mediated by specialized pro-resolving mediators (SPM) which target specific resolutive G-protein coupled receptors (GPCR) expressed by different immune cells and participates in the return to tissue homeostasis after injury without being immunosuppressive (5). Defects in this process fuels chronic inflammation and contributes to carcinogenesis and exacerbates tumor growth. In contrast, administration of SPM controls tumor growth in several preclinical models (4).

The GPCR ChemR23, encoded by the gene *CMKLR1*, contributes to both initiation and resolution of inflammation, depending on the ligand that binds to it, in various acute or chronic inflammation models (6, 7). Two ligands were described for ChemR23, the pro-resolutive lipidic resolvin E1 (RvE1) and the chemoattractant protein Chemerin, encoding by the gene *RARRES2*. ChemR23 is mainly expressed on the innate immune cells including monocytes, macrophages, dendritic cells and some Natural Killer (NK) cells as well as on adipocytes and endothelial cells (8–11). Its activation by RvE1 has been reported to increase phagocytic activity of macrophages and to induce a proresolutive phenotype (12). In addition to RvE1, Chemerin, widely recognized as an adipokine particularly abundant in inflammatory fluids (9), binds ChemR23 at the site of inflammation (8), where it promotes recruitment of monocytes/macrophages and their adhesion to extracellular matrix proteins (13). Chemerin-derived peptides processed on the site of inflammation have been reported to have anti-inflammatory effects that contribute to inflammation resolution in ChemR23 dependent manner (14, 15). The biology of ChemR23 is complex as its activation can trigger distinct inflammatory or resolving pathways, which determine the outcome of inflammation.

Macrophages critically orchestrate chronic inflammation and related diseases. They display high intrinsic plasticity and adaptability based on epigenetic regulation relying on various signals emanating from the microenvironment. Beyond the canonical M1/M2 macrophage phenotype dichotomy, single-cell analysis has recently emphasized macrophage diversity during differentiation and activation processes especially during cancers (16, 17). Even though macrophage phenotypes in cancers appeared to be part of a continuum contributing to either pro- (mainly immunosuppressive) or anti-tumor activities, they mainly exhibit a protumoral M2-like phenotype favored by their reeducation by cancer cells, that often reduces cancer therapy efficacy (18). Reprogramming tumor-associated macrophages (TAM) has thus been viewed as therapeutic opportunities to treat cancers (7).

Although ChemR23-mediated activation in macrophages during inflammation has been recently documented (19), its role in cancer progression has not been yet fully explored. The objective of this study was to evaluate the effect of an agonist antibody directed against ChemR23 (αChemR23) (19) in the pathological context of cancers. We thus first evaluated ChemR23 expression in our models of GM-CSF and M-CSF differentiated macrophages. αChemR23 effect *in vitro* on M-CSF macrophages has been explored at transcriptomic, protein and functional levels. We then extended our study to cancer contexts, using TAM-like models (differentiation of monocytes in tumor cell supernatants), focusing on BC and MPM, in which TAM exert a decisive impact on tumor progression (20).

Finally, *in vivo* activation of ChemR23 with αChemR23 was evaluated in immunocompetent orthotopic murine model of triple-negative BC on tumor growth, metastasis and survival after tumor resection. Altogether, our data indicate that ChemR23 regulates macrophage phenotype and cancer-related inflammation in tumors and that targeting ChemR23 may contribute to control tumor progression and metastasis.

## Materials and methods

### ChemR23 agonist

The pro-resolutive agonist αChemR23 mAb was produced and purified by OSE Immunotherapeutics as previously reported and characterized (19). The hIgG1 control mAb (clone MOTA-hIgG1) was produced in parallel by Evitria (Switzerland).

### Cells

Human monocytes were freshly isolated by magnetic sorting from PBMC of healthy volunteers following the manufacturer’s protocol (classical monocyte isolation kit, Miltenyi Biotec). The MPM cell line Meso13 was established from the pleural fluid of a MPM patient (21), characterized for its karyotype (GSE 134349) and for their mutational status using targeting sequencing (*CDKN2A*del, *CDKN2B*del). Cal51 cell line, derived from metastatic site of a triple negative BC (pleural effusion), and the murine triple-negative BC 4T1-luc2 cell line were purchased from DSZM (Braunschweig, Germany) and ATCC, respectively. These cell lines were cultured in complete RPMI-1640 (Gibco) or DMEM 4.5g/l Glucose media (Gibco) supplemented with 2mM L-glutamine, 100IU/mL penicillin, 0.1mg/mL streptomycin (Gibco) and 10% heat-inactivated fetal calf serum FCS (Gibco) at 37°C and 5% CO2 atmosphere.

### Analysis of tumor gene expression profiling

All RNAseqv2 samples from the The Cancer Genome Atlas (TCGA)-MESO dataset (n=87 patients) and BRCA dataset (n=1997) are available on the Broad’s Genome Data Analysis Centre (http://gdac.Broad-institute.org/). Gene expressions as RNA-seq by expectation maximization values (RSEM values) were analyzed. Clinical data for these samples were downloaded from FireBrowse (http://firebrowse.org; version 2018_02_26 for MESO and BRCA). The Breast Cancer Gene-Expression Miner v4.8 (http://bcgenex.ico.unicancer.fr/BC-GEM/GEM-Accueil.php?js=1) was used to analyze *CMKLR1* expression in BC with macrophage markers.

### Macrophage polarization

Monocytes were seeded, in 12-wells plates, at 0.5x10^6^ cells/ml in 2.5ml of complete medium supplemented with GM-CSF at 20ng/ml (Cellgenix, 001412-050) or with M-CSF at 50ng/ml (Isokine, 01-A0220). To obtain TAM-like macrophages, monocytes were differentiated with undiluted supernatant from MPM (Meso13) or BC (Cal51) cell lines as previously described (22). Macrophages were kept in culture for 3 days at 37°C and 5% CO2 atmosphere and analyzed after treatment as described below at days 4-5.

### ChemR23 signaling

M-CSF differentiated macrophages were starved for 4h in medium then stimulated with hIgG1 or αChemR23 (10µg/ml) for different times. Cells were lysed with 200µl of RIPA (Sigma-Aldrich) buffer 1X supplemented with protease inhibitor cocktail (Fast protease inhibitor, Sigma-Aldrich) and phosphatase inhibitors (Phos STOP, Roche). Cell lysates were centrifuged for 25min at 800g at 4°C to remove debris and the supernatants were stored at -80°C. Proteins were quantified using Bradford assay (Interchim). 7µg of proteins were loaded in 4-20% Mini-PROTEAN^®^ TGX™ Precast Protein Gel (#4561093, Biorad) and then transferred onto a nitrocellulose membrane. After 2h of saturation in 5% milk/TBS-Tween 0,05%, the membranes were incubated with primary antibodies anti–phospho(Thr202/Tyr204) - ERK1/2 (p44/42 MAPK) (#4370, Cell Signaling) (1:1000), anti- ERK1/2 (p44/42 MAPK) (#9102S, Cell Signaling) or anti–phospho(Ser473) -AKT (#4051S, Cell Signaling) (1:1000), anti-AKT (#4691S, Cell Signaling) or anti-Actin M(AB1501 Millipore) for 1h at room temperature. Proteins were incubated with Goat anti-Rabbit (#111-001-003) or Goat anti-Mouse (115-035-008) secondary antibodies (Jackson ImmunoResearch) for 1h at room temperature and then revealed with the Immobile Western Chemiluminescent HRP Substrate (WBKLS0500, EMD Millipore). The data were analyzed with the Fusion FX device (Vilber).

### Immunophenotyping by flow cytometry

Macrophages were detached using phosphate-buffered saline (PBS)-EDTA at 4°C for 30min and labeled with live/dead marker FVS510 (BD, 564406) for 30min at 4°C in PBS. After washing, the cells were labeled with anti-CD14 APC-Vio770 (clone REA599, Miltenyi Biotec), anti-HLA-DR FITC (clone L243, BD), anti-CD16 BV421 (clone 3G8, Biolegend), anti-CD163 AF647 (clone GHI/61, BD) and anti-ChemR23 PE (clone 84939, R&D system) antibodies for 30 min at 4°C in PBS-0,01% bovine serum albumin (BSA) (Sigma Aldrich). IgG1k-AF647 (clone MOPC-21, BD), REA control-APCvio770 (clone REA293, Miltenyi Biotec), IgG3-PE (clone 133316, R&D system), IgG1,k-FITC (MOPC-21, BD) and IgG1,k-BV421 (clone MOPC-21, Biolegend) isotypes were used as controls. Analysis was performed by flow cytometry using FlowJo v10 software. The results are expressed as the ratio of the median fluorescence obtained with the specific antibody to the median fluorescence obtained with the corresponding isotype (RFMI=Ratio of Median fluorescent intensity).

For tSNE data projection, M-CSF differentiated macrophages were incubated with 10µg/ml αChemR23 or hIgG1 antibodies for 24h and processed for flow cytometry staining as mentioned above with the following antibodies: anti-CD14 APC (clone MSE2, Biolegend), anti-CD163 (clone GHI/61.1, Miltenyi), anti-CD192 BV605 (clone K036C2, Biolegend), anti-CD206 BB515 (clone 19.2, BD), anti-CD274 PE (clone MIH1, BD), anti CD45 APC R700 (clone HI30, BD), anti-CD209 BV421 (clone DCN46, BD), anti-CD80 BUV395 (clone L307.4, BD), anti-CD16 BUV737 (clone 3G8, BD), anti HLA-DR BV711 (clone L243, Biolegend).

Analysis of the data was performed using FlowJo v10 software.

### Chemokine secretion assays

After 3 days in culture for differentiation, macrophages were incubated with 10µg/ml αChemR23 or hIgG1 antibodies for 24h. Then, cells were stimulated with 200ng/ml lipopolysaccharide (LPS) (Sigma-Aldrich) and the supernatant were collected after 24h for TNF-α, IL-1β, IL-1RA, IL-6, IL-10, IL-12p70, IL-12p40, CCL17, IL-23 and IP-10 quantification using the LEGENDplex Human M1/M2 macrophages panel (BioLegend) according to the manufacturer’s recommendations.

### Transcriptomic analysis

After 3 days in culture, macrophages were incubated with 10µg/ml of αChemR23 or hIgG1 antibodies for 24h. Then, cells were stimulated with 200ng/ml lipopolysaccharide (LPS) (Sigma-Aldrich) for 6h and cells were lysed using the RLT buffer (Qiagen) supplemented by 1% β-mercaptoethanol (Sigma-Aldrich). Total RNA was isolated using the RNeasy Mini Kit according to the manufacturer’s protocol (Qiagen).

For RT-qPCR experiments, 0.5µg of total RNA was reverse transcribed using MMLV reverse transcriptase (Invitrogen). PCR reactions were performed using QuantiTect Primer Assays (Qiagen) and RT^2^ Real-time SYBR-Green/ROX PCR mastermix (Qiagen) and carried out using QuantStudio™ Real-Time PCR system 3 (ThermoFisher). *RPLP0*, *ACTB* and *GAPDH* gene expression were used as internal standards.

For Nanostring analysis, gene expression was quantified with the NanoString nCounter platform using 50ng of total RNA in the nCounter Human (594 genes) or Mouse (561 genes) Immunology Panel (NanoString Technologies). The code set was hybridized with the RNA overnight at 65°C. RNA transcripts were immobilized and counted using the NanoString nCounter Sprint. Normalized expression data were analyzed with the nSolver software. Standardized not log2-transformed counts were used for differential gene expression analysis with the R package DESeq2 (23). Genes were ranked in order of differential expression and *P* value score. Gene set enrichment analysis was performed with the GSEA software with 1000 permutations. Score signatures of transcriptomic analysis (single sample GSEA score) were obtained calculating a gene set enrichment score per sample as the normalized difference in empirical cumulative distribution functions of gene expression ranks inside and outside the gene set with the R package GSVA.

### ChemR23 immunolabelling of human breast and mesothelial tumors

Human tumor specimens were collected from 7 treatment-naive patients with invasive BC after surgical resection at the Institut de Cancérologie de l’Ouest, Nantes/Angers France, and from 3 patients with MPM. Informed consent was obtained from enrolled patients and protocol was approved by Ministère de la Recherche (agreement n°: DC-2012-1598) and by local ethic committee (agreement n°: CB 2012/06) or through the biological resource center (CHU Nantes, Hôtel Dieu, Tumorothèque, Nantes, France BRIF: BB-0033-00040, transfer number 122C366) for patients with BC tumors or MPM, respectively. Three-micrometers-thick tissue sections of formalin-fixed, paraffin-embedded (FFPE) breast cancers were treated with protease for 4min (Protease 1, Ventana, 760-2018) to achieve epitope retrieval. Samples were then incubated during 32min at 37°C with the anti-ChemR23 antibody at a dilution of 1:200 (clone 1A7, mouse monoclonal, Origene) and with the anti-Iba1 antibody at a dilution of 1:1000 (clone EPR16588, rabbit monoclonal, Abcam). Protein expression was detected with an OptiView DAB IHC Detection Kit (Roche Diagnostics, 760-700), optimized for automated immunohistochemistry (Benchmark GX stainer, Ventana Medical Systems, Roche Diagnostics). ChemR23 and Iba1-immunolabelled macrophages were quantified on 10 High Power Fields (HPFs, Fields of 1590 μm^2^ at 400X magnification) in tumor and peritumor (comprising a thickness of 200µm around the periphery of the tumor) areas.

### Phagocytosis assay

For induction of tumor cell apoptosis, the BC cell line Cal51 was treated with 70nM paclitaxel (Sigma-Aldrich) plus 100nM of the BCLxL antagonist A1331852 (SelleckChem) for 24h, as previously described in (24). Meso13 cells were treated with 1.6 µg/ml cisplatin (Merck) and 80 µM pemetrexed (Sigma-Aldrich) for 72h, as previously described in (16). Apoptotic and control untreated cells were then harvested, washed and labelled with 1µg/ml of the pH sensitive dye pHrodo^®^ (Incucyte pHrodo Red cell labelling Kit for phagocytosis, Sartorius, 4649) following the manufacturer’s protocol. In parallel, differentiated macrophages were seeded at 10^4^ cells in 96-well plates in 200µl of complete medium and treated with 10µg/ml αChemR23 or hIgG1 antibodies for 24h at 37°C. Then, labelled apoptotic or untreated cancer cells were added to macrophages at a ratio of 1:5 for 24h and phagocytosis was measured by live cell imaging using Incucyte^®^ technology (Sartorius). Phagocytosis was quantified using fluorescence intensity (“Total integrated intensity”) in integrated software.

### Polyclonal T cell proliferation assay

Differentiated macrophages treated with 10 µg/ml αChemR23 or hIgG1 antibodies for 24h were stimulated with 200 ng/ml LPS for additional 24h, supernatants (conditioned media) were collected and centrifuged to remove cell debris, aliquoted and stored at -80°C.

Human T lymphocytes were freshly isolated by negative magnetic sorting (EasySep Human T cell isolation kit, Stemcell technologies) from PBMC of healthy volunteers following the manufacturer’s protocol. Isolated T lymphocytes were labelled with 0.5 µM of cell proliferation dye (CPD) following the manufacturer’s protocol (eBioscience™ Cell proliferation dye efluor 670, ThermoFischer). 0.1x10^6^ labelled T lymphocytes were activated with CD3/CD28 beads at a ratio 1:40 or 1:100 for 5 days (Dynabeads Human T-Activator CD3/CD28, ThermoFischer) in 96-well U-bottom plates and treated with conditioned media from macrophages, IL-2 at 75 U/ml or IL-10 at 50 ng/ml (R&D system). After 5 days in culture, T lymphocytes were washed twice with PBS and labeled with live/dead dye APC-H7, anti-CD3 FITC (clone REA163), anti-CD4 PE (clone RPA-T4) and anti-CD8 BV421 (clone RPA-T8) antibodies. Analysis was performed by flow cytometry using DIVA software.

### Preclinical tumor model

Animal housing and surgical procedures were conducted according to the guidelines of the French Agriculture Ministry (APAFIS 8629-2017011915305978) and were approved by the local animal ethics committee (CEEA-PdL n°6 (pour Comité d’éthique en expérimentation animale_Pays de la loire n°6)). BALB/c mice were purchased from Janvier Laboratories and kept in the UTE Nantes SFR Bonamy animal facility. 4T1-luc2 cells were harvested and resuspended in PBS for animal inoculation. Metastasis spreading was monitored using bioluminescence (Biospace Imager) by injecting 100µl of D-luciferin (Interchim) at 33.33mg/ml through intraperitoneal route. 4T1-luc2 cells (0.25x10^6^/mouse in PBS) were injected into the fat pad of mammary gland of 8-week-old BALB/c female mice (day 0). Mice were treated with αChemR23 or hIgG1 mAbs intraperitoneally at 1mg/kg 3 times a week for 3 weeks from day 7 to day 28. At day 13, primary tumors were surgically removed from mammary glands. Tumor spreading was analyzed by overall survival and by *in vivo* bioluminescence on lungs every week using a bioimager. Mice were euthanized when critical endpoints were reached according to criteria defined by ethical committees and lungs were harvested for further experiments including transcriptomic analysis (Nanostring technology and RT-qPCR) and flow cytometry, as previously described.

### Statistical analysis

The two data groups were compared with the nonparametric test Mann-Whitney. To assess the significance of the effect of αChemR23 compared with hIgG on macrophages, the Wilcoxon paired t-test was used. The log-rank test was used to compare survival times between the two groups. Errors bars represent standard errors of mean (SEM). The symbols correspond to a *P*-value inferior to *0.05, **0.01, ***0.001, ****0.0001. All statistical analyses were performed using GraphPad Prism software (version 8.0).

## Results

### ChemR23 expression and activation in human M-CSF macrophages

**Figure 1:**
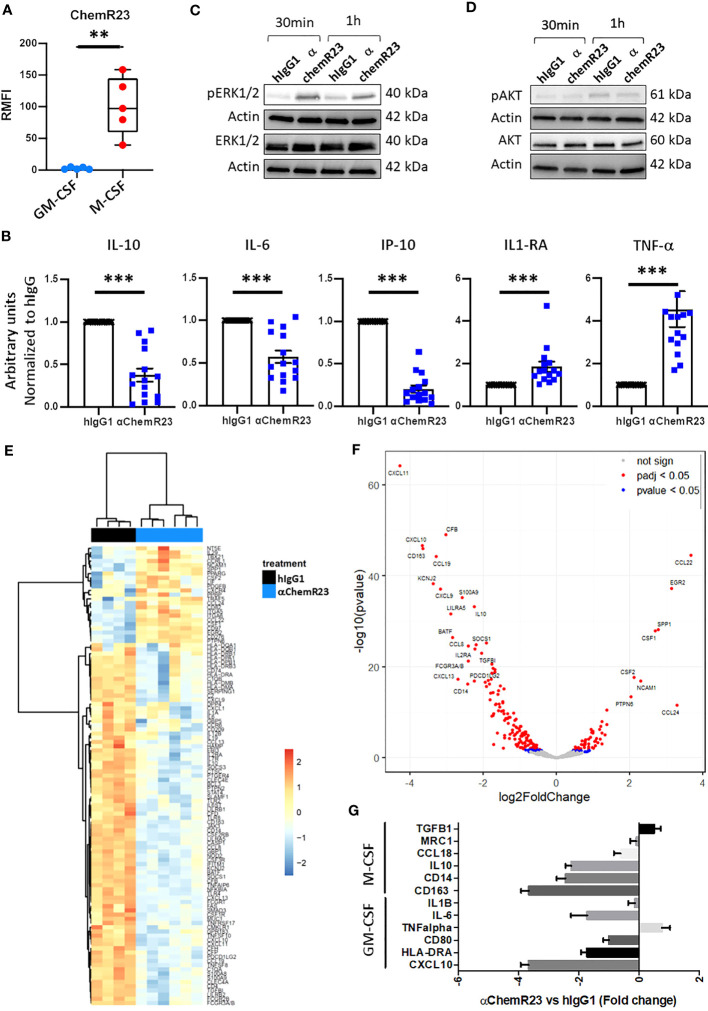
ChemR23 expression and activation in M-CSF macrophages. **(A)** Human monocytes from 5 healthy donors were differentiated *in vitro* using GM-CSF (20ng/ml) or M-CSF (50ng/ml) for 3 days, then ChemR23 expression was analyzed by flow cytometry. RFMI=Ratio of Median fluorescent intensity **, p < 0.01. **(B)** M-CSF macrophages were treated with αChemR23 or control hIgG1 for 24h. Then, cells were stimulated with LPS (200 ng/ml) for 24h and supernatants were collected. The indicated cytokines were quantified using multiplex ELISA in the corresponding supernatants. n= 15. *, p < 0.05; **, p < 0.01; ***, p < 0.001. pERK1/2(Thr202/Tyr204) **(C)** and pAKT(Ser473)/AKT **(D)** ChemR23 signaling assessed by immunoblot in M-CSF macrophages treated with either 10µg/ml of αChemR23 agonist antibody or with control hIgG1 for 30min or 1h. **(E-G)** M-CSF differentiated macrophages were treated with αChemR23 agonist or control hIgG1 for 24h followed by LPS stimulation (200ng/ml) for 6h. mRNA were extracted and analyzed using NanoString Technology (Human immunology panel). **(E)** Heatmap of genes differentially expressed between αChemR23 agonist versus control hIgG1 treated macrophages from 4 and 6 donors respectively with a p value adjusted ≤ 0,05 and absolute log2 fold change ≥ 1. **(F)** Volcano plot showing differentially expressed genes following αChemR23 agonist treatment, and genes with absolute value of the log2 fold change ≥ 2 and p value adjusted ≤ 0.05 are highlighted with their gene code name. **(G)** Graphic showing variation of expression of a panel of genes specific of GM-CSF or M-CSF macrophages. n=4-6.

We first evaluated the expression of ChemR23 in M-CSF or GM-CSF differentiated human macrophages. M-CSF is a cytokine usually secreted by tumor cells and found in malignant tissues (25). M-CSF macrophages present phenotypic and functional characteristics different from M2a-c macrophages (26–28), but close to those of TAM as described in ovarian cancer (28) thus representing an interesting model of macrophages with immunomodulatory properties in the context of a malignancy. In contrast, using GM-CSF usually found elevated in the context of inflammation or immune response (29) leads to macrophages with proinflammatory properties however different from macrophages obtained using IFN-γ -/+ LPS corresponding to a particular context of host defense (30). As previously observed, M-CSF macrophages expressed a significantly higher level of CD14 and CD163 (27), and a significantly lower level of CD80 and HLA-DR than GM-CSF macrophages (**Figure S1**) (28). GM-CSF expressed a higher level of CD206 and CD192 than M-CSF macrophages whereas the latter expressed a significantly higher level of CD16 than GM-CSF macrophages (**Figure S1**). No evident differences in CD209, CD45 and CD274 expression were observed on both model of macrophages (**Figure S1**). As expected, GM-CSF macrophages secreted significant higher level of IL-12p70 and TNFα, and significant lower level of IL-10, IL-1RA and IP-10 than M-CSF macrophages (**Figure S2A**) (30, 31). No differences in the secretion of IL-6 and IL-1β was observed between M-CSF and GM-CSF macrophages (**Figure S2A**). M-CSF macrophages expressed significantly more ChemR23 than GM-CSF macrophages (**Figure 1A**). To evaluate the biological activity of ChemR23 agonist antibody, previously reported to activate the pro-resolutive signaling of ChemR23 (19), on macrophages, we measured cytokine secretion. Treatment with αChemR23 mAb significantly decreased IL-10, IP-10 and IL-6 secretion, and significantly increased TNFα and IL-1RA secretion while not changing IL-12p70 and IL-1β secretion of M-CSF macrophages (**Figures 1B**, **S3A**). On GM-CSF macrophages, αChemR23 treatment led to a significant decrease of IP-10 secretion only (**Figure S3B**). These results suggest that αChemR23 mAb mainly impact M-CSF macrophages functions thus, we focused our study on this macrophage subtype.

To confirm that αChemR23 triggered ChemR23 activation in M-CSF macrophages, we studied intracellular signaling pathways. Treatment by αChemR23 mAb induced phosphorylation of ERK1/2 within 30min or 1h (**Figure 1C**) without triggering AKT pathway (**Figure 1D**). These results demonstrate the capacity of the agonist mAb to induce an intracellular signaling pathway in M-CSF macrophages.

In order to better characterize the impact of ChemR23 activation on M-CSF macrophages, we performed a transcriptomic analysis using NanoString Technology with the Human immunology panel. Unsupervised hierarchical clustering representation shows that ChemR23 triggering by the agonist antibody induced strong gene expression modifications compared to hIgG1 control (**Figure 1E**). Volcano plot representation (**Figure 1F**) indicates in red points significant (padj < 0.05) gene expression modifications with 53 induced and 137 repressed genes on a total of 594 analyzed genes, and genes with an absolute log2 fold change ≥ 2 are highlighted by their gene code name (8 induced and 19 repressed). Using discriminating genes between M1-like and M2-like signatures (32), we evidenced a decrease of both M2-associated markers *CCL18*, *IL10*, *CD14*, *CD163*, *IL6* expression as well as M1-associated ones *CD80*, *HLA-DRA* and *CXCL10*, encoding IP-10, and a slight increase of *TNFalpha* and *TGFB1* (M1 associated) (**Figure 1G**). In contrast *MRC1* and *IL1B* were not affected. Interestingly, we found that *IDO1* expression, coding for Indoleamine 2,3-dioxygenase-1 a potent T-cell immunosuppressive enzyme, was also reduced by anti-ChemR23 treatment on M-CSF macrophages (**Figure S4A**). These results argue for the induction of an intermediate phenotype different from the GM-CSF or M-CSF-induced macrophages polarization following ChemR23 activation.

### ChemR23 activation modulates the expression of macrophage surface markers

**Figure 2:**
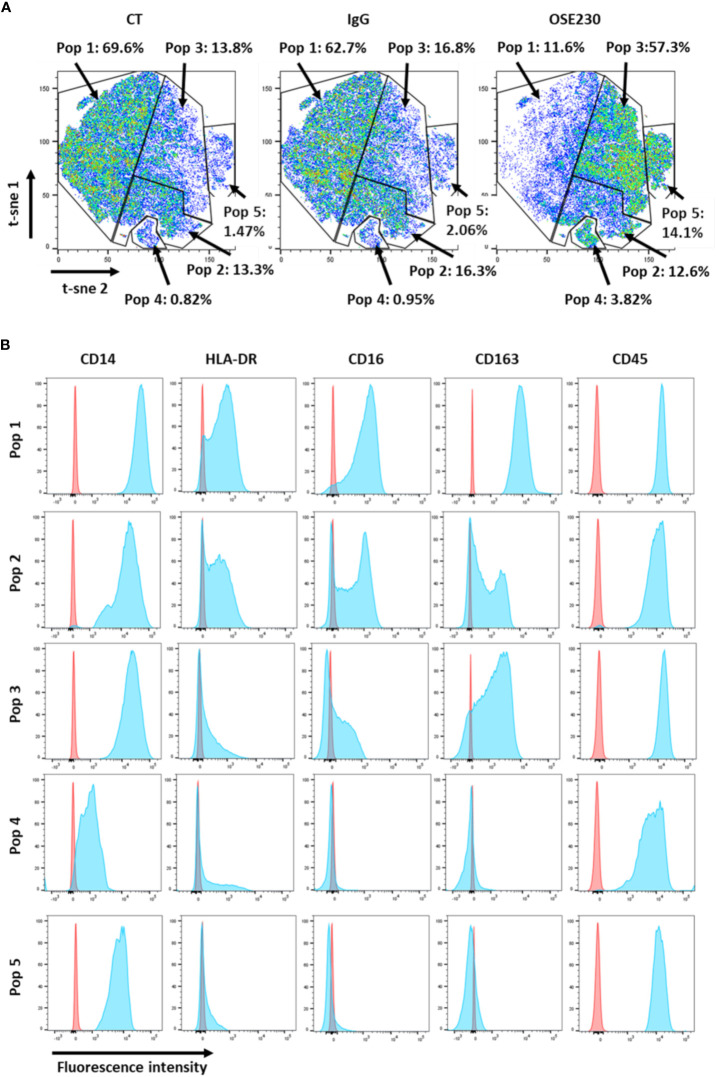
αChemR23 agonist modifies M-CSF macrophages cell surface markers expression. M(M-CSF) macrophages were treated for 24h with αChemR23 or control hIgG1 and cells were collected and labelled with a panel of 10 antibodies including: CD209, CD163, CD14, CD16, CD206, HLA-DR and CD45 and analyzed using flow cytometry. **(A)** Macrophage subpopulations represented using t-SNE. **(B)** Phenotype of the different macrophage subpopulations impacted by treatment with αChemR23. Experiments were performed using monocytes from 3 different healthy donors and results include data from the 3 donors.

In order to study the impact of αChemR23 mAb on macrophage phenotype, we measured the expression of 10 cell surface markers, CD14, CD163, CD192, CD206, CD274, CD45, CD209, CD80, CD16, HLA-DR, using flow cytometry. According to our transcriptomic study, the expression of CD14, CD16, CD163 and HLA-DR was decreased whereas CD206 was not modified (**Figure S5**). tSNE representation (**Figure 2A**) shows that the decrease of marker expression was not homogenous and led to a redistribution of macrophage subpopulations. Indeed, as examples, we observed an increase of CD14 high, CD163 med, CD16 low and HLA-DR low cells (16.8% to 57.3%) (**Figure 2B**, pop 3), CD14 high, CD163 -, CD16 -, and HLA-DR low cells (2.06% to 14.1%) (**Figure 2B**, pop 5) and CD14 low, CD163 -, CD16 -, and HLA-DR low cells (0.95% to 3.82%) (**Figure 2B**, pop 4), and a decrease of CD14 high, CD163 high, CD16 high and HLA-DR med (62.7% to 11.6%) (**Figure 2B**, pop 1) macrophage subpopulations. CD14 high, CD163 med, CD16 med and HLA-DR med macrophage subpopulation was weakly impacted by αChemR23 mAb (16.3% to 12.6%) (**Figure 2B**, pop 2).

### ChemR23 activation modulates macrophage activities

**Figure 3:**
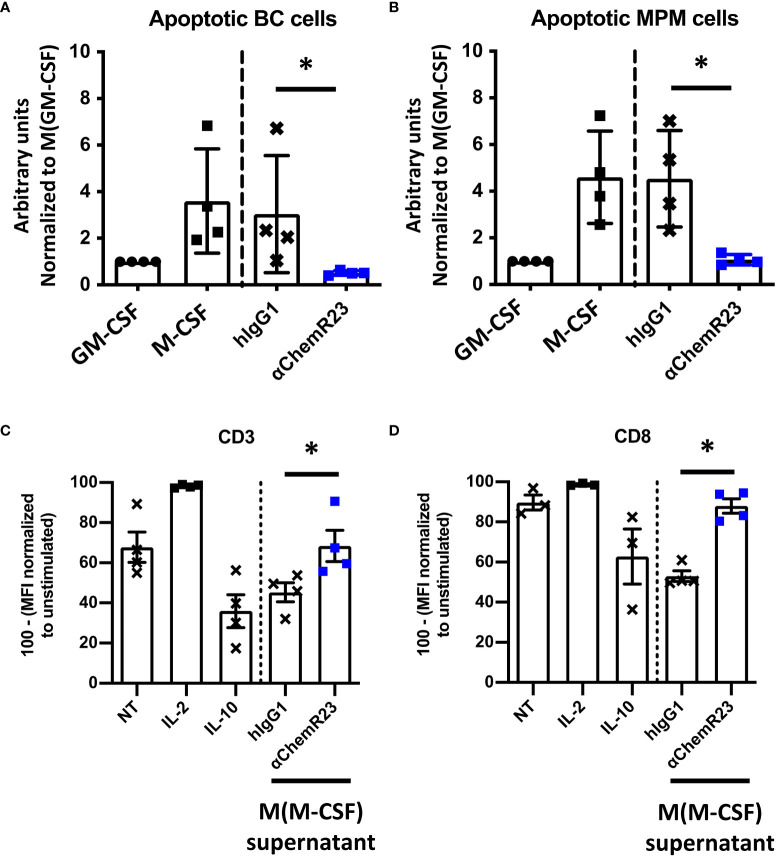
αChemR23 modulates functional properties of M-CSF macrophages. For phagocytosis assay, **(A)** breast cancer or **(B)** mesothelioma (MPM) apoptotic cells were incubated with untreated GM-CSF macrophages or M-CSF macrophages (left panels) and M-CSF macrophages (M(GM-CSF)) pretreated or not with human hIgG1 control or αChemR23 mAb for 24h (right panels). Efferocytic activity was quantified by live-cell imaging (Incucyte^®^). Results represent the maximum fluorescence intensity normalized to M(GM-CSF) used as reference. n=4. *, p < 0.05. **(C, D)** For T cell proliferation assay, CPD stained CD3+ T-cells were incubated for 5 days with CD3/CD28 beads and either IL-2 (75 U/ml), or IL-10 (50 ng/ml), or supernatants of M(M-CSF) macrophages (MΦ) treated with αChemR23 agonist (10µg/ml) or hIgG1 control (10µg/ml) and T-cells were assessed by flow cytometry. Frequency of non-fluorescent proliferating cells was evaluated after gating on CD3+ cells **(C)** and after gating on CD8+ cells **(D)** and normalized to the unstimulated condition. n=4. *, p < 0.05.

Since M-CSF macrophages were deeply impacted in response to ChemR23 activation, we further extended our study on two macrophage main functions, phagocytosis and T-cell proliferation modulation. Phagocytic activity of macrophages during inflammation is fundamental for its resolution, we thus evaluated macrophage phagocytosis capacity, i.e. their ability to phagocyte apoptotic tumor cells, here BC and MPM apoptotic cells, following ChemR23 activation. We found that phagocytosis capacity was, as expected, higher in M-CSF (3.5 fold) compared to GM-CSF macrophages and only occurred towards apoptotic cancer cells (**Figures S6A, B**). Treatment of M-CSF macrophages with αChemR23 induced a significant reduction of their capacity to internalize apoptotic cancer cells (**Figures 3A, B**). No effect of the agonist mAb was observed on GM-CSF macrophages phagocytosis activity (**Figures S6C, D**). These results illustrate that ChemR23 activation by the antibody deeply affects human M-CSF macrophages including modification of their capacity to phagocyte apoptotic tumor cells.

Furthermore, macrophages can also exert their immunomodulatory function through regulation of T-cell proliferation, hence we incubated T lymphocytes from PBMC from 3 healthy donors with macrophages supernatants previously incubated with ChemR23 agonist antibody or with the isotype control antibody. T-cell activation was induced by stimulation with CD3-CD28 beads for 5 days, then cell-proliferation dye dilution was measured by flow cytometry in total CD3+ T cells (**Figure 3C**) or CD3+ CD8+ T lymphocytes (**Figure 3D**) and CD3+ CD4+ T lymphocytes (**Figure S7**). As positive control, IL-2 potently increased T cell proliferation while IL-10 decreased T-cell proliferation at the concentration used in this assay. Of interest, we found that the supernatants from M-CSF macrophages also reduced the proliferation of human T-cell but this immunosuppressive effect was reversed when macrophages were priorly incubated with the agonist anti-ChemR23 mAb (**Figures 3C, D**). Of note, this effect was not significant on CD4 T-cells (**Figure S7**).

These results demonstrate that ChemR23 triggering by an agonist mAb strongly modifies human M-CSF macrophages polarization as illustrated by the profound transcriptomic, phenotypic, cytokines secretion and immune functions modifications.

### Expression of *CMKLR1* in BC and MPM correlates to TAM markers

**Figure 4:**
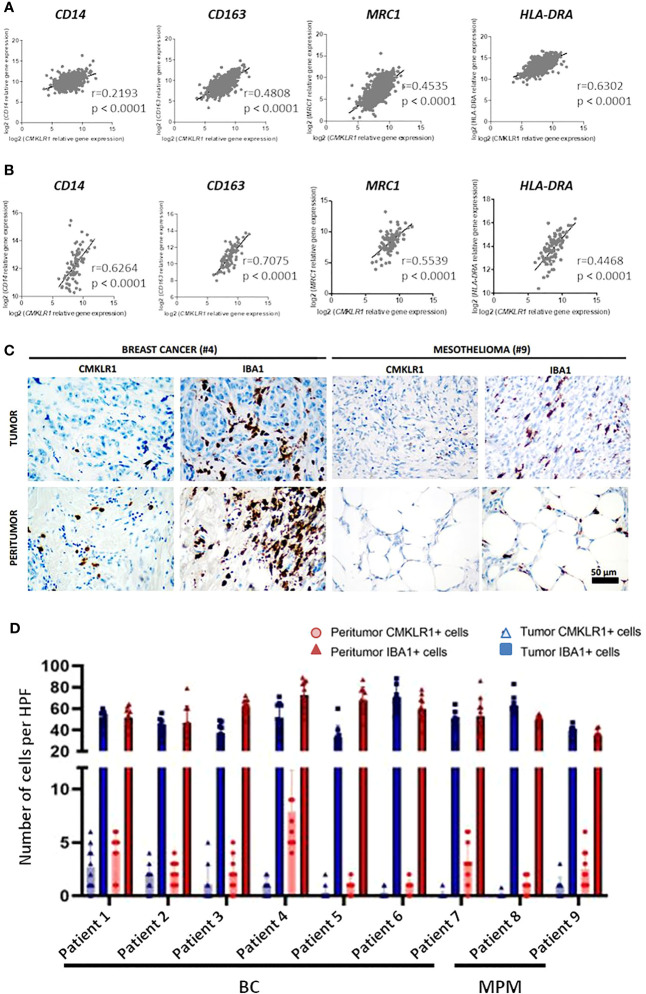
*CMKLR1* expression in breast cancer and mesothelioma tumors correlates to TAM markers and ChemR23 is expressed in *in vitro* TAM-like models. Correlation of *CMKLR1* expression with macrophage markers *CD14*, *CD163*, *MRC1* and *HLA-DRA* in breast cancers (n=1034) **(A)** and mesothelioma (n=87) **(B)** tumors using transcriptomic data from the TCGA database. **(C)** IHC staining of ChemR23 and Iba1 in breast tumors and MPM from 9 patients in tumor and peritumoral areas. **(D)** Quantification of ChemR23 IHC staining by HPF (high power field, 1590μm^2^) for each patient.

Regarding the involvement of M2-like macrophages in cancers (33), we extended our study to two malignancies in which the presence of these cells was described as associated with the severity of the disease, breast cancer (34) and malignant pleural mesothelioma (35). We first studied the expression of *CMKLR1* in BC and MPM, using the dataset of The Cancer Genome Atlas (TCGA), where these pathologies appear to express high level of *CMKLR1* among 37 cancers analyzed (**Figure S8A**). We observed that *CMKLR1* was expressed in all subtypes of BC or MPM with a significant lower expression in luminal breast tumors (**Figure S8B**) and epithelioid mesothelioma tumors (**Figure S8C**). Importantly correlation analyses showed significant co-expression between *CMKLR1* and genes specific of tumor associated macrophages (TAM) such as *CD14*, *CD163*, *MRC1* and *HLA-DRA* in BC (**Figure 4A**) and MPM (**Figure 4B**). Of note, IHC analysis performed in breast and mesothelioma tumors from patients indicated that ChemR23 expression could be detected in a part of macrophages evidenced by Iba1 staining in tumor or in peritumoral areas (**Figures 4C, D**). Using a public database of single cell RNA sequencing performed on 8 breast tumors ((36), http://panmyeloid.cancer-pku.cn/), we observed that *CMKLR1* is mainly expressed in a macrophage subpopulation with a high expression of *CD14*, *CD163* and *HLA-DRA*, and with a low expression of *CD80* and *CD86*. Additionally, expression of *CMKLR1* was not observed in macrophage subpopulations expressing high levels of *HLA-DRA*, *CD80* and *CD86* (**Figure S9A**). The high expression of *CMKLR1* in macrophages expressing high level of *CD14* and *CD163* was confirmed using two additional public databases (**Figures S9B, C**) (37, 38). Based on these observations, we evaluated the expression of ChemR23 on macrophages obtained by incubation of monocytes from healthy donors with BC or MPM cancer cell culture supernatants, as model of TAM (TAM-like), and compared to the one of M-CSF and GM-CSF macrophages. **Figure S2B** shows TAM-like exhibited a phenotype close to the M-CSF one with high expression of CD14 and CD163, low/med expression of HLA-DR, high/med secretion of IL-10 and IP-10, and low secretion of TNFα and IL12p70 (**Figure S2A**). Importantly, TAM-like macrophages expressed significantly higher levels of ChemR23 compared to GM-CSF macrophages and similar levels of ChemR23 compared to M-CSF macrophages (**Figure S2B**).

Altogether, these data illustrate that ChemR23 is expressed by TAM in human tumors. Moreover, our *in vitro* models of TAM seem to reproduce, at least partially, pathological situation.

### Activation of ChemR23 in TAM-like macrophages also modulates their phenotype

**Figure 5:**
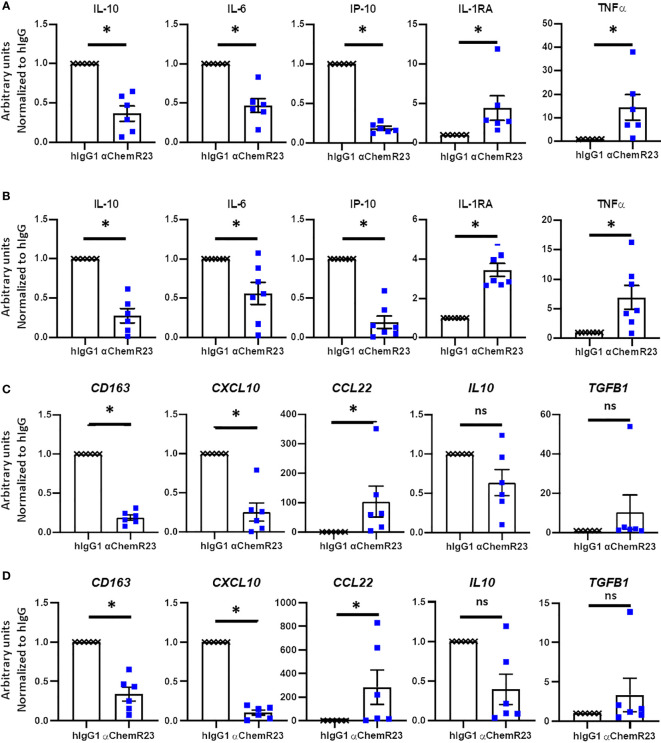
αChemR23 agonist modulates TAM-like macrophages cytokine secretion and mRNA expression profiles. Monocytes from healthy donors were incubated with conditioned media of BC cells **(A, C)** or MPM cells **(B, D)** for 3 days were treated with 10µg/ml of αChemR23 agonist or control hIgG1 for 24h. For cytokine quantification **(A, B)**, supernatants were collected after 24h stimulation with LPS (200ng/ml) then the indicated cytokines were quantified using multiplex ELISA in the supernatants. n= 6. *, p < 0.05. For mRNA analyses **(C, D)**, cells were lysed after 6h stimulation with LPS (200ng/ml) then mRNA were extracted and gene expressions were measured using RT-PCR. n= 5. *, p < 0.05; **, p < 0.01.

In order to appreciate the potential of ChemR23 triggering on human macrophages, we used our models of BC and MPM TAM-like macrophages. As observed for M-CSF macrophages, ChemR23 activation by the agonist antibody significantly reduced IL-10, IP-10 and IL-6 secretion, and significantly induced TNFα and IL-1RA secretion while not affecting IL-12p70 in both BC (**Figures 5A**, **S10A**) and MPM TAM-like (**Figure 5B**, **S10B**). Using RT-PCR, we also observed a significant decrease of *CD163* and *CXCL10* genes, a tendency for *IL-10* expression to decrease, and a significant increase of *CCL22* and a tendency for *TGFB1* genes to increase in both BC (**Figure 5C**) and MPM (**Figure 5D**) TAM-like macrophages. As in M-CSF macrophages, we observed that *IDO1* expression tended to be reduced by anti-ChemR23 treatment on MPM TAM-like macrophages, but not significantly due to the limited number of donors (decreased expression of *IDO1* in 4 out of 5 donors, p=0.0625), (**Figure S4B**). This tendency was not obtained in BC TAM-like where *IDO1* expression was poorly detectable compared to MPM (**Figure S4C**). These data indicate that ChemR23 receptor strongly controls macrophage phenotype and that its activation promotes a different macrophage polarization with a phenotype potentially less inflammatory and less immunosuppressive. Moreover, the tumor microenvironment could drive angiogenesis by modulating macrophage activity, thus to address the possibility of the αChemR23 antibody modulation on this function in macrophages, we studied the expression of two well-known genes involved in angiogenesis and vessel remodeling, *VEGFA* and *MMP9* (**Figure S11A, B**). First, we observed a difference of expression of *VEGFA* and *MMP9* by TAM-like depending on the cell culture supernatant was from BC or MPM. Indeed, BC TAM-like highly expressed *VEGFA* compared to MPM TAM-like (**Figure S11A** right panel) and MPM TAM-like expressed more *MMP9* than BC TAM-like (**Figure S11B** right panel). ChemR23 activation with the agonistic antibody decreased the *VEGFA* expression by BC TAM-like (**Figure S11A**), the *MMP9* expression by MPM TAM-like and tended to decrease *MMP9* expression by BC TAM-like (**Figure S11B**).

**Figure 6:**
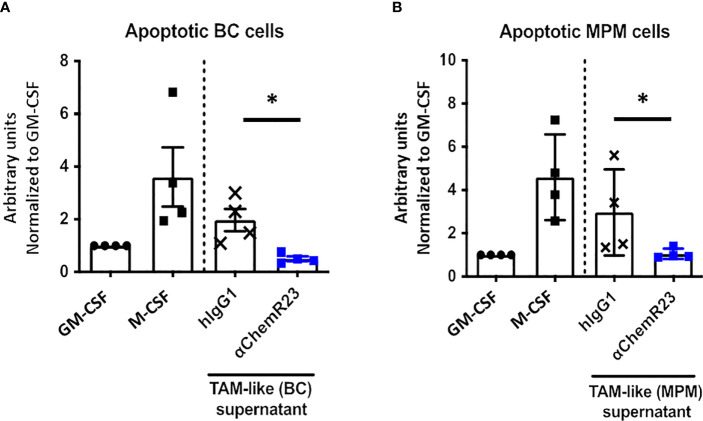
αChemR23 decreased phagocytic properties of TAM-like macrophages. Phagocytosis of apoptotic **(A)** breast cancer (BC) or **(B)** mesothelioma (MPM) cell lines by untreated GM-CSF and M-CSF macrophages or TAM-like macrophages pretreated with αChemR23 or hIgG1 control (10µg/ml) for 24h and quantified by live-cell imaging (Incucyte^®^). Results represent the maximum fluorescence intensity normalized to GM-CSF macrophages condition. n=4. *, p < 0.05.

We further assessed the effect of αChemR23 mAb on TAM-like phagocytosis activity. BC and MPM TAM-like macrophages displayed a phagocytosis activity similar to the one of M-CSF macrophages (**Figures S7A, B**). As previously observed for M-CSF macrophages, a reduction of phagocytosis activity in BC and MPM TAM-like by 4 and 3 folds, respectively, was observed following treatment with αChemR23 (**Figures 6A, B**).

### Anti-ChemR23 mAb treatment alters metastasis development and immune microenvironment in murine breast cancers

**Figure 7:**
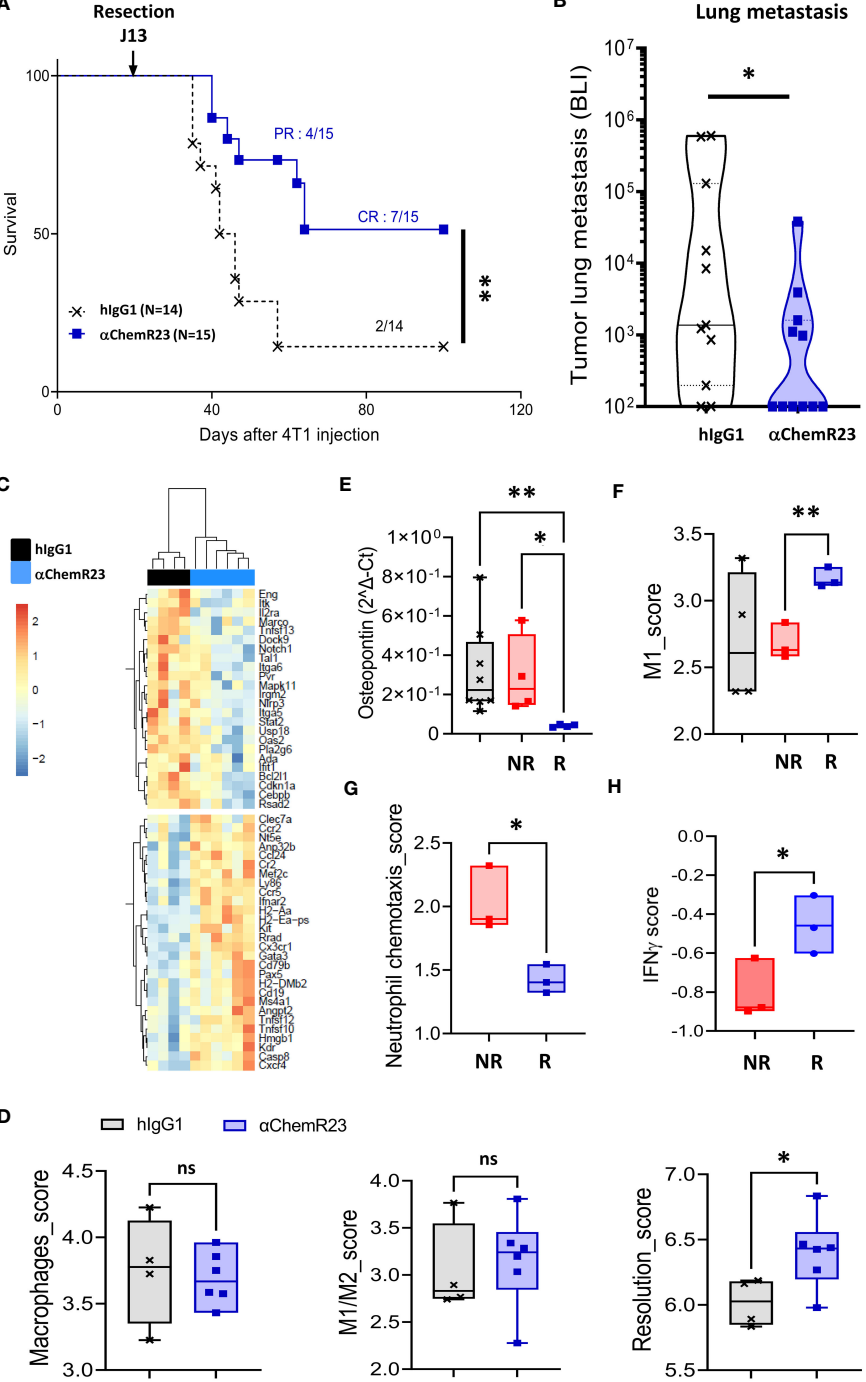
αChemR23 monotherapy increases survival and reduces metastasis in a murine orthotopic breast tumor model. Survival **(A)** and lung metastasis count by bioluminescence (BLI) **(B)** of 4T1-luc2 bearing mice treated i.p. with hIgG1 control or αChemR23 mAb (1mg/kg, 3 times a week from d7 to d28). Representative heatmap of clustered differential gene expression **(C)** and scores of transcriptomic analysis **(D)** of lungs in the αChemR23 (blue; n=7) or isotype control (black; n=4) treated 4T1-luc2-bearing mice that have been resected at d13 using the Nanostring mouse PCIP panel. **(E)** Osteopontin mRNA expression, measured using RT-PCR, in lung tumors of mice treated with hIgG1 control (black, n=8) or αChemR23 non responders (red, n=4) or αChemR23 responders (blue, n=4) mAb. **(F)** M1 score signature of transcriptomic analysis of lungs from αChemR23 and isotype hIgG1 (black; n=4 or 8) mAb-treated mice. **(G)** Neutrophil chemotaxis scores and **(H)** IFNγ scores of transcriptomic analysis of lungs in the αChemR23 (red for non-responders n =3-4; blue for responders n=3) mAb-treated mice. ns, non-significant; *, p < 0.05; **, p < 0.01. NR, non-responders n=3-4; R, responders n= 3-4.

αChemR23 antibody being able to activate human as well as mouse ChemR23 receptor (**Figure S12**), we further investigate the interest to target ChemR23 in cancers, we evaluated agonist anti-ChemR23 mAb treatment *in vivo* on BC progression using the orthotopic syngeneic triple-negative 4T1-luc2 model. Mice treated with ChemR23 agonist antibody in monotherapy initiated 7-8 days after tumor implantation, displayed no significant tumor growth modification compared to hIgG1 control at the dose tested (**Figure S13**). To closer mimic clinic situation for most patients with BC, tumor resection 13 days after tumor cell injection was performed in mice treated with anti-ChemR23 mAb 7-8 days after tumor implantation and 5-6 days before tumor resection (corresponding to the peak of metastatic risk), in order to reprogram ChemR23 expressing cells before the tumor resection and to evaluate the subsequent effect on long term metastatic development. Our results indicate that mice survival was significantly increased in the treated group resulting in 7/15 mice still alive (complete response) 100 days after tumor injection while only 2/14 in the control group survive at long-term (**Figure 7A**). In addition, 4 mice in the treated group had a partial response that improved their survival. Importantly, this observation correlated with significant decreased lung metastasis detection in treated mice using *in vivo* bioluminescence (**Figure 7B**) compared to the control group. No lung metastasis 30 days after tumor cell injection could be detected in 55% of treated mice compared to 18% in the control group. Transcriptomic analysis of lung metastasis (using the murine immunology panel from Nanostring technology) indicated that αChemR23 treatment induced significant gene expression modifications compared to hIgG1 control as shown in unsupervised hierarchical clustering representation in **Figure 7C** and showed, in particular, an increase of the resolution score in αChemR23 mAb treated group, although macrophages or M1/M2 scores were not modified (**Figure 7D**). Importantly, based on the metastasis associated gene expression *SPP1*, coding for Osteopontin, as a marker of the 4T1-luc2 metastatic cells, we defined 2 mice groups corresponding to responders, low *SPP1* expression, and non-responders, high *SPP1* expression (**Figure 7E**). Using Nanostring data, we observed that M1 score signature was strongly increased in the responding mice compared to the non-responding ones (**Figure 7F**). This was confirmed by the increase of CCR7 positive macrophages detected in metastasis microenvironment by flow cytometry (**Figure S14A**). Moreover, neutrophil chemotaxis score significantly decreased in responders (**Figure 7G**, **S14B**), as previously observed in inflammatory disease model (19). Finally, using the IFNγ score that predicts the response to T-cell immunotherapy (39) and is correlated to the NK and T cell activity, we found a significant increase of the IFNγ score in responder animals compared to non-responders even at this very early stage of metastasis development (**Figure 7H**).

Altogether, these results illustrate that an anti-ChemR23 agonist mAb can modify *in vivo* ChemR23 positive cells and limits distant metastasis development in monotherapy.

## Discussion

Dysregulation of macrophage biology during carcinogenesis fuels cancer progression and resistance to treatments. In this study, we explored the opportunity to reprogram TAM by targeting the receptor ChemR23 using an agonist pro-resolutive antibody (19). Our data indicate that ChemR23 was preferentially expressed on M-CSF and TAM-like macrophages compared to GM-CSF macrophages. We report that the expression of ChemR23 coding gene, *CMKLR1*, in breast and mesothelioma tumors positively correlates to the expression of TAM markers such as *CD14*, *CD163*, *MRC1* and *HLA-DRA*, in agreement with ChemR23 detection restricted to macrophages in tumors from patients. We show that triggering ChemR23 signaling with the αChemR23 mAb deeply modulated these macrophages at the transcriptomic, phenotypic, cytokine secretion and functional levels with a resulting less inflammatory and less immunosuppressive profile different from the M1 and M2 dichotomy. Interestingly, *in vivo* treatment in monotherapy with this mAb, in an aggressive triple-negative breast cancer model, significantly reduced tumor dissemination and increased overall survival in correlation to TAM modulation in metastatic niche.

In this work, we show the higher expression of ChemR23 in M-CSF macrophages compared to GM-CSF macrophages. Previously, few studies described the expression of ChemR23 in human macrophages. In 2015, Herová et al. described an expression of ChemR23 in IFNγ or LPS stimulated macrophages higher than in IL-4 or IL-13 stimulated macrophages (40). In 2016, Peyrassol et al. showed the expression of this receptor at the surface of M-CSF macrophages as in our study however without comparing to a model of pro-inflammatory macrophages (41). Regarding the experimental condition used in these two studies, the comparison is difficult and could only suggest that depending on the protocol, ChemR23 expression should be different. However, using scRNASeq data from breast tumors, we mainly observed an expression of *CMKLR1* in a subset of TAM C1QC^+^ that expressed usual M2 signature, including high expression of CD14 and CD163, whereas *CMKLR1* expression was low in a subset of TAM *ISG15*+ that expressed classical M1 signature (36). These results confirm our observations and support that our models of macrophages reproduce, at least in part, TAM from tumors.

We report here that agonist αChemR23 potently decreased LPS stimulated-IL-10, IP-10 and IL-6 secretion and expression of cell surface markers such as CD163, CD14 and HLA-DR by either M-CSF or TAM like macrophages in *in vitro* experiments, as well as angiogenic modulators (*VEGFA*, *MMP9*). In the opposite, TNFα and IL-1RA, a natural inhibitor of IL-1β, were significantly increased, suggesting that ChemR23 activation led to a hybrid macrophage phenotype, as further confirmed by deeper transcriptomic analysis using Nanostring technology and flow cytometry characterization. Importantly, functional analysis of these macrophages indicated that their immunosuppressive properties towards T cell proliferation were also reduced. Of specific interest, IL-10 that is considered as a potent immunosuppressive cytokine in tumors thus promoting their immune escape, was strongly down-regulated after ChemR23 activation, as much as *IDO1* expression and to a lesser extent *ARG1* expression (data not shown) depending on the macrophage differentiation, and then could contribute to the effect observed. Other cytokines such as IP-10 that was also significantly repressed upon ChemR23 activation, may also contribute to alleviate tumor progression since it also modulates angiogenesis (42). Altogether, these data argue for a profound reprogramming of human M-CSF or TAM-like macrophages, upon exposure to agonist anti-ChemR23 mAb, to a phenotype different from the M1-M2 dichotomy with less inflammatory, less immunosuppressive functions and less phagocytosis activity.

The macrophage phenotype obtained after αChemR23 treatment seems also different from the one of resolutive macrophages previously reported since characterized by an increase of secretion of IL-10 and TGF-β, and of efferocytosis activity (43–45). The differences observed in our study can be attributed to the pathological context and the type of macrophages studied. Indeed, in the majority of cases, inflammatory macrophages were used (M1, M2a, M2b and M2c) whereas in our study, we used M-CSF macrophages, named M2d, with a specific and different transcriptomic profile close to TAM *in vivo* (26, 46). Indeed, M-CSF was described to be produced by cancer cells, including MPM cells (22), and cells from the TME and therefore highly present in malignant tissues (25). Moreover, cytokine secretion was measured here after LPS stimulation to activate macrophages (31), a situation completely different from the one used in previous study where macrophages were not stimulated and studied in a context of inflammatory pathology (19). Finally, resolutive macrophage have a high efferocytosis activity, however in this study we have studied phagocytosis of apoptotic tumour cells but not of apoptotic neutrophils (efferocytosis). The description of human resolutive macrophages remains also poorly documented and requires further studies to clearly define this population (47). A recent single cell RNA Sequencing study in mice has identified several populations of phagocytic or non-phagocytic macrophages coexisting within the same tissue (48). Moreover, several subpopulations of phagocytic macrophages exist, one of which is described as “satiated” after pro-resolving lipid mediators treatment such as resolvin E1 (44). The particularities of these macrophages are a low expression of CD11b and a low efferocytosis and phagocytosis activity. Macrophages resulting from αChemR23 treatment could be closer to one of them, and might be characterized as “satiated” or “post-resolving” (44, 49).

Of major importance, our results revealed that ChemR23 activation by the agonist antibody led to decreased occurrence of lung metastasis and increased survival in a preclinical model of aggressive triple-negative breast tumors, when primary tumors were resected. Interestingly, stimulating resolution of inflammation using AINS or resolvins before tumor surgery decreased micrometastases in multiple tumor resection preclinical models through induction of T cell response (50) which is in accordance with the increase of IFNγ score observed in αChemR23 responding mice. Our results strongly suggest that *in vivo* ChemR23 activation modulated tumor cell metastasis onset that may rely on decrease of dissemination and/or modification of the metastatic niche. Complementary experiments would be necessary to further dissect which cellular and molecular events are in play during the formation of this process. Importantly, Sulciner and colleagues previously reported that using proresolving lipids such as RvE1 in preclinical models of tumor growth, that relied on chemotherapy-induced cell debris, counteracted tumor progression through stimulation of macrophage phagocytic activity and decrease of their proinflammatory cytokine (TNFα, IL-6) release that occurred in a ChemR23-dependent process (4). The other ligand of ChemR23, chemerin, was previously shown to suppress breast cancer growth through recruitment of mainly NK dependent immune effectors in the tumor microenvironment (51). However, it may also exert a protumoral activity directly on tumor cells or through tumor associated mesenchymal or endothelial cells (52). In addition, chemerin is released by cancer-associated myofibroblasts in mammary tumors where it contributes to cancer cell invasion (53). Previously reported characterization of the agonist pro-resolutive anti-ChemR23 mAb have however excluded a chemerin-like activity of the antibody based on different signaling induction and opposite effect in chemoattraction assay (19).

In conclusion, in line with previous reports demonstrating the therapeutic interest to promote inflammation resolution during cancer treatments, our results also argue that targeting ChemR23 using an agonist antibody may improve cancer evolution in limiting metastasis occurrence. Using human M-CSF and TAM-like macrophages, we showed that targeting ChemR23 deeply affected their phenotype and functions leading to a less inflammatory and immunosuppressive profile. These changes could contribute to improve anti-tumor immune response and then, disease outcome. Our results fuel the proof of concept that modulating TAM phenotype to harness their antitumor potential, would improve cancer therapy efficacy.

## Data availability statement

The datasets presented in this study can be found in online repositories. The names of the repository/repositories and accession number(s) can be found below: NCBI via accession ID GSE218330.

## Ethics statement

Informed consent was obtained from enrolled patients and protocol was approved by Ministère de la Recherche (agreement n°: DC-2012-1598 and DC-2011-1399) and by local ethic committee (agreement n°: CB 2012/06) or through the biological resource center (CHU Nantes, Hôtel Dieu, Tumorothèque, Nantes, France BRIF: BB-0033-00040, transfer number 122C366) for patients with breast tumors or mesothelioma, respectively. The patients/participants provided their written informed consent to participate in this study. The animal study was reviewed and approved by Comité d’éthique en expérimentation animale_Pays de la loire n°6.

## Author contributions

NP, SB-N, and CB conceived the study and the experimental setup, analyzed the experiments, wrote the manuscript with input from all authors and are responsible for the overall content of this manuscript; ML and VG performed the experiments and the data analysis and wrote the manuscript; CM produced αChemR23 antibody; IG and VG performed the analysis of nanostring experiments; SD performed RT-PCR and analysis; ML, JF, and VD performed cytometry experiments on human cells and analysis; AD performed western-blot; FC carried out histology experiments and analysis; ML and VG performed the mouse experiments; CM and SN carried out the follow-up of the *in vivo* experiments and biological studies; and PJ gave critical input to the writing of the manuscript. All authors contributed to the article and approved the submitted version.

## Supplementary material

Supplementary Figure 1 Characterization of GM-CSF and M-CSF macrophages. Monocytes were incubated with GM-CSF (20 ng/ml) or M-CSF (50ng/ml) for 72h and analyzed by flow cytometry using a panel of 10 antibodies. n= 3. *, p < 0.05; **, p < 0.01; ***, p<0.001.

Supplementary Figure 2 Characterization of MPM and BC TAM-like macrophages. Monocytes were incubated with GM-CSF (20 ng/ml), M-CSF (50ng/ml) or conditioned media from breast (TAM-like (BC)) or mesothelioma (TAM-like (MPM)) cell lines for 3 days and stimulated with LPS (200ng/ml) for 24h. **(A)** Supernatants were collected and the indicated cytokines were analyzed by ELISA. n= 5. *, p < 0.05; **, p < 0.01. **(B)** Cells were analyzed by flow cytometry for the expression of the indicated markers. n= 5. *, p < 0.05; **, p < 0.01.

Supplementary Figure 3 αChemR23 agonist modulates macrophage’s secretion profile (raw data related to ). The indicated cytokines were quantified by ELISA in the supernatants of **(A)** M-CSF (n=15) or **(B)** GM-CSF (n=4) differentiated macrophages treated with hIgG1 or αChemR23 (10µg/ml) for 24h followed by LPS stimulation for 24h. *, p < 0.05; **, p < 0.01; ***, p < 0.001, ****, p < 0.001.

Supplementary Figure 4 Effect of aChemR23 antibody on *IDO1* expression in M-CSF and TAM-like macrophages. M-CSF and MPM cell culture supernatant differentiated macrophages were treated with αChemR23 agonist or control hIgG1 for 24h followed by LPS stimulation (200ng/ml) for 6h. mRNA were extracted and expression of *IDO1* was analyzed using **(A)** Nanostring Immunology Panel (M-CSF macrophages, n=6) or **(B)** RT-PCR (MPM TAM-like macrophages, n=5). **(C)** Differential expression of *IDO1* in BC and MPM TAM-like. *, p < 0.05; **, p < 0.01, ****, p < 0.001.

Supplementary Figure 5 αChemR23 agonist modifies M-CSF macrophage cell surface markers expression. M-CSF macrophages were treated for 24h with αChemR23 or control hIgG1 and cells were collected and labelled with a panel of 10 antibodies including: CD209, CD163, CD14, CD16, CD206, HLA-DR and CD45 and analyzed using flow cytometry Graphics presented are representatives of experiments performed using monocytes from 3 different healthy donors.

Supplementary Figure 6 Macrophage phagocytosis. GM-CSF, M-CSF and TAM-like macrophages were respectively incubated with untreated or apoptotic **(A)** BC or **(B)** MPM cell lines and phagocytosis was measured every hour by live cell imaging (Incucyte^®^), n=3. Phagocytosis of **(C)** BC or **(D)** MPM apoptotic cells by GM-CSF macrophages pretreated with αChemR23 or control hIgG1 for 24h and quantified by live-cell imaging (Incucyte^®^). Results represent the maximum fluorescence intensity normalized to GM-CSF macrophages condition, n=4.

Supplementary Figure 7 CD4 T-cell proliferation regulation by supernatants of M-CSF macrophages treated with αChemR23 agonist. For T cell proliferation assay, CPD stained CD3+ T-cells were incubated for 5 days with CD3/CD28 beads and either IL-2 (75 U/ml), or IL-10 (50 ng/ml), or supernatants of M(M-CSF) macrophages (MΦ) treated with αChemR23 agonist (10µg/ml) or hIgG1 control (10µg/ml) and T-cells were assessed by flow cytometry. Frequency of non-fluorescent proliferating cells was evaluated after gating on CD3+/CD4+ cells and normalized to the unstimulated condition. n=4. *, p < 0.05.

Supplementary Figure 8
*CMKLR1* expression in breast cancer and mesothelioma tumors. **(A)** Expression of *CMKLR1* using transcriptomic data of breast cancer (n=1034) **(B)** and MPM tumor samples (n=87) **(C)** from the TCGA database. *CMKLR1* expression in **(B)** breast tumor subtypes (BRCA) and in **(C)** mesothelioma (MPM) tumor subtypes. EM: Epithelioid mesothelioma, BM: Biphasic mesothelioma, SM: Sarcomatoid mesothelioma. *, p < 0.05; **, p < 0.01; ****, p < 0.0001. **(D)**

Supplementary Figure 9
*CMKLR1* expression in breast cancer tumors using single cell RNA sequencing database. **(A)** Expression of *CMKLR1*, *CD14*, *CD163*, *HLA-DRA* and *CD80* in breast tumors (n=8) monocyte/macrophage subpopulations using single cell RNA sequencing database from http://panmyeloid.cancer-pku.cn/. Left panel, boxplot of gene signature expression level per cell calculated with the geometric mean of all gene expression levels. Right panel, heatmap representation of the median gene expression levels in monocyte/macrophage subgroups. **(B)** tSNE representation of the expression of *CMKLR1, CD14* and *CD163* in five triple negative breast cancers using single cell RNA sequencing database from https://singlecell.broadinstitute.org/single_cell/study/SCP1106/stromal-cell-diversity-associated-with-immune-evasion-in-human-triple-negative-breast-cancer. **(C)** tSNE representation of the expression of *CMKLR1, CD14* and *CD163* in 26 breast cancers (11 ER+, 5 HER2+ and 10 TNBCs) using single cell RNA sequencing database from https://singlecell.broadinstitute.org/single_cell/study/SCP1039/a-single-cell-and-spatially-resolved-atlas-of-human-breast-cancers.

Supplementary Figure 10 αChemR23 agonist effect on TAM-like macrophages’ cytokine secretion profile (raw data related to ). Human monocytes were differentiated using breast **(A)** mesothelioma **(B)** cell culture supernatants for 3 days. TAM-like macrophages were treated with 10µg/ml of αChemR23 agonist or human control IgG1 for 24h and stimulated with LPS (200 ng/ml) for 24h. Supernatants were collected and the indicated cytokines were quantified using multiplex ELISA in the supernatants. n= 6-7. *, p < 0.05; **, p < 0.01.

Supplementary Figure 11 αChemR23 agonist affects expression of genes involved in immune response modulation and angiogenesis. BC or MPM cell culture supernatant differentiated macrophages were treated with αChemR23 agonist or control hIgG1 for 24h followed by LPS stimulation (200ng/ml) for 6h. mRNA were extracted and expression of *VEGFA* **(A)** and *MMP9* **(B)** was analyzed using RT-PCR. n=5. *, p < 0.05; **, p < 0.01.

Supplementary Figure 12 αChemR23 agonist activity on human, mouse and cynomolgus ChemR23. Recombinant human, cynomolgus and mouse ChemR23 was produced and purified by G-CLIPS Biotech company (Labège, France) after HEK transfection with a plasmid encoding the ChemR23 sequence with a fluorescent conformational dye on the cytoplasmic end of the TM6 and using an appropriate formulation buffer. Receptor activation can be assessed by monitoring the shift delta of between the TM6 opening or closing. After production, ChemR23 recombinant was encapsulated in appropriated lipid micelles to better recapitulate the conformation of the receptor on a cell-surface membrane. **(A)** Dose response of human, mouse and cynomolgus ChemR23 activation curves, **(B)** table of kinetics parameters of activation (Bmax and Kd) with αChemR23 antibody.

Supplementary Figure 13 αChemR23 monotherapy effect on primary tumor growth in murine breast tumor model. **(A)** Primary tumor volume (mm^3^) measured in the 4T1-luc2 bearing mice treated i.p. with a control mAb or αChemR23 mAb (1mg/kg, 3 times a week from d7 to d28).

Supplementary Figure 14 αChemR23 monotherapy strongly modifies the metastasis microenvironment by controlling the macrophage function. **(A)** Percent of CCR7+ M1 macrophages into lungs in the αChemR23 (isotype hIgG1 (black; n=4 or 8); red for non-responders n=3-4; blue for responders n=3-4) mAb-treated mice. **(B)** Representative heatmap of clustered differential gene expression of lungs in the αChemR23 (red for non-responders n=3-4; blue for responders n=3) mAb-treated mice.
