# Supplementary material for: ChemR23 activation reprograms macrophages toward a less inflammatory phenotype and dampens carcinoma progression
Source: Front Immunol. 2023 Jul 19;14:1196731. doi: 10.3389/fimmu.2023.1196731 (PMC10396772; doi:10.3389/fimmu.2023.1196731)
Supplement: Supplementary Figure 1 — Characterization of GM-CSF and M-CSF macrophages. Monocytes were incubated with GM-CSF (20 ng/ml) or M-CSF (50ng/ml) for 72h and analyzed by flow cytometry using a panel of 10 antibodies. n= 3. *, p < 0.05; **, p < 0.01; ***, p<0.001. [file Presentation_1.pptx]

## Slide 1
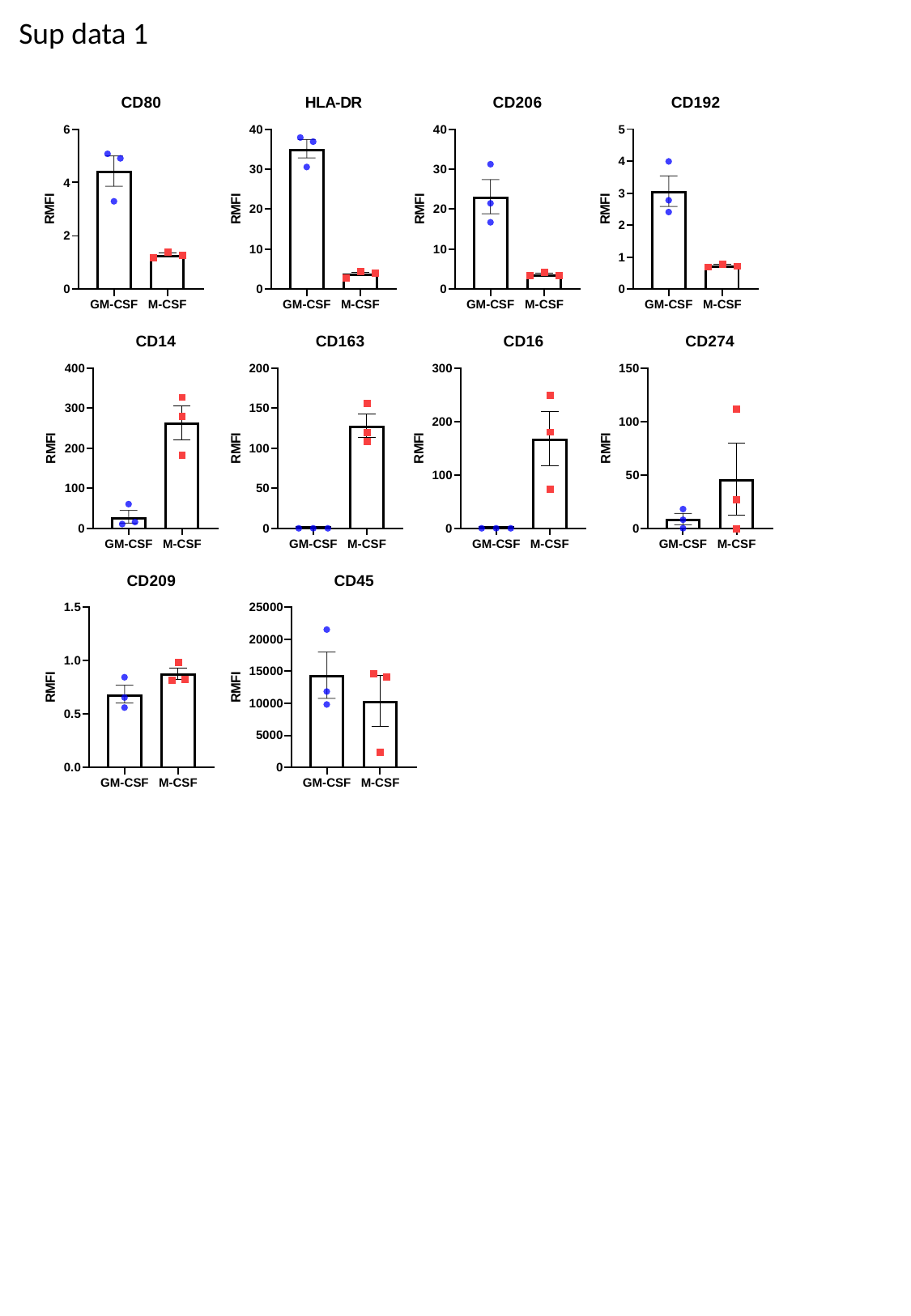

Sup data 1

## Slide 2
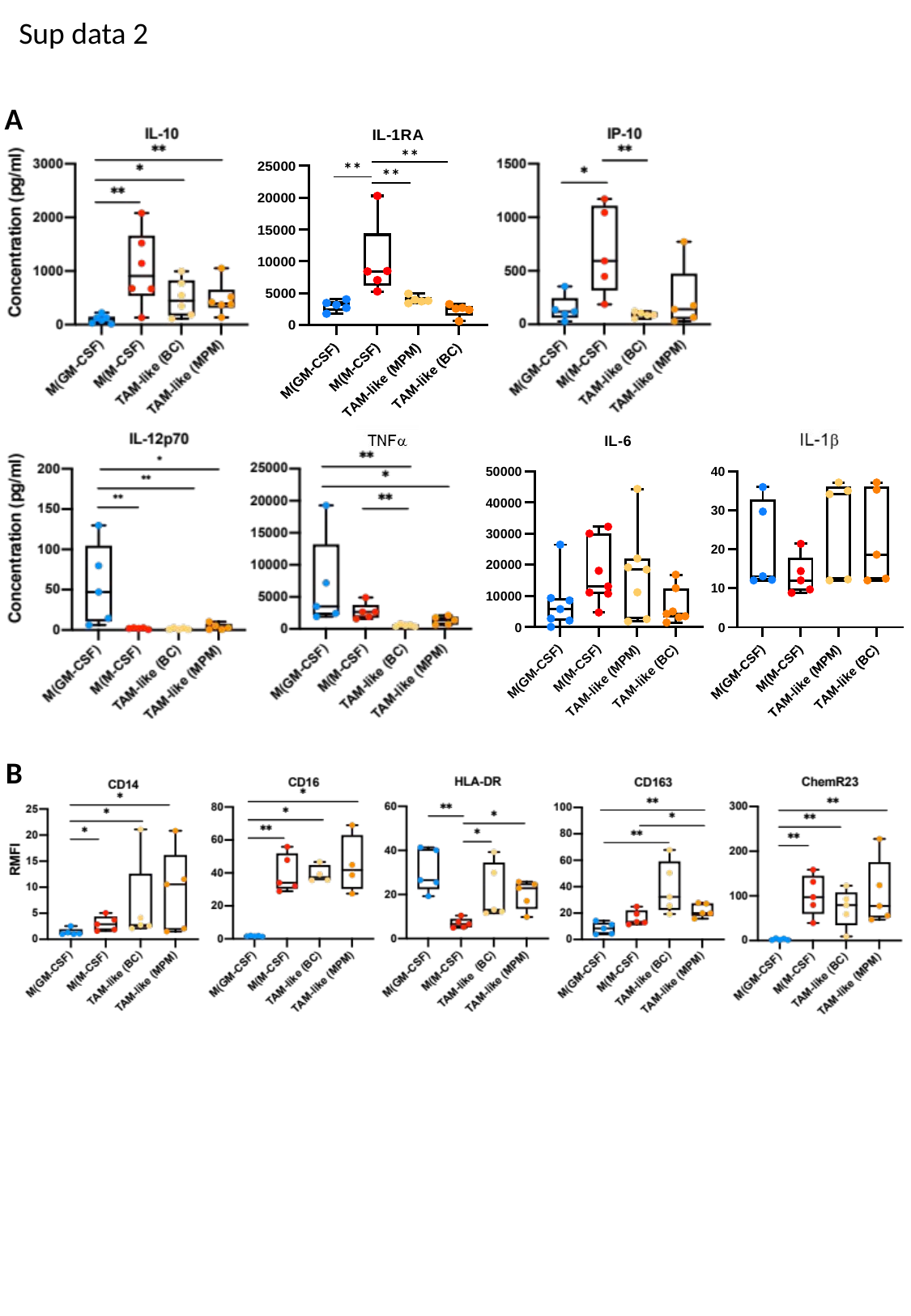

Sup data 2
A
**
**
**
B

## Slide 3
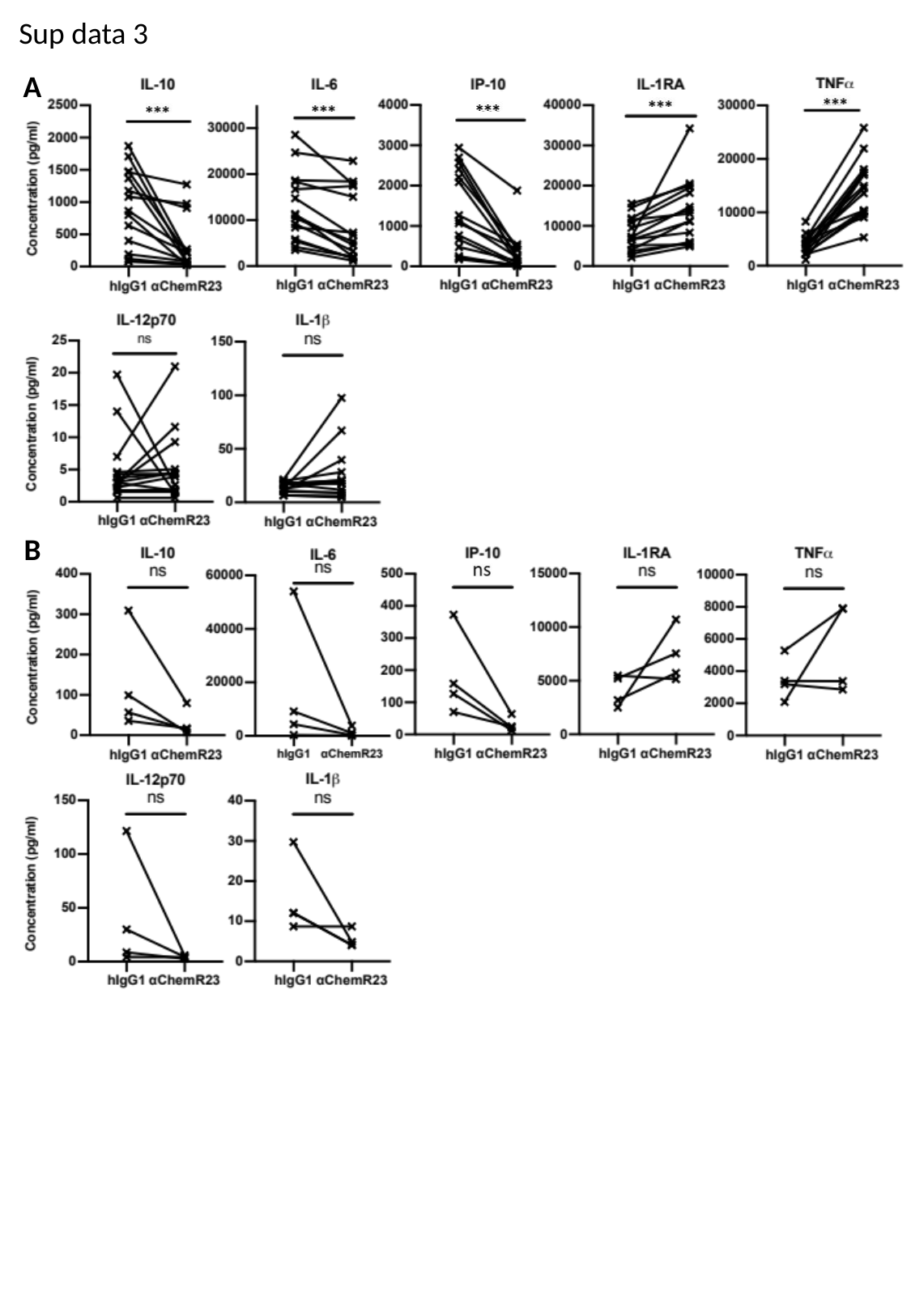

Sup data 3
A
B

## Slide 4
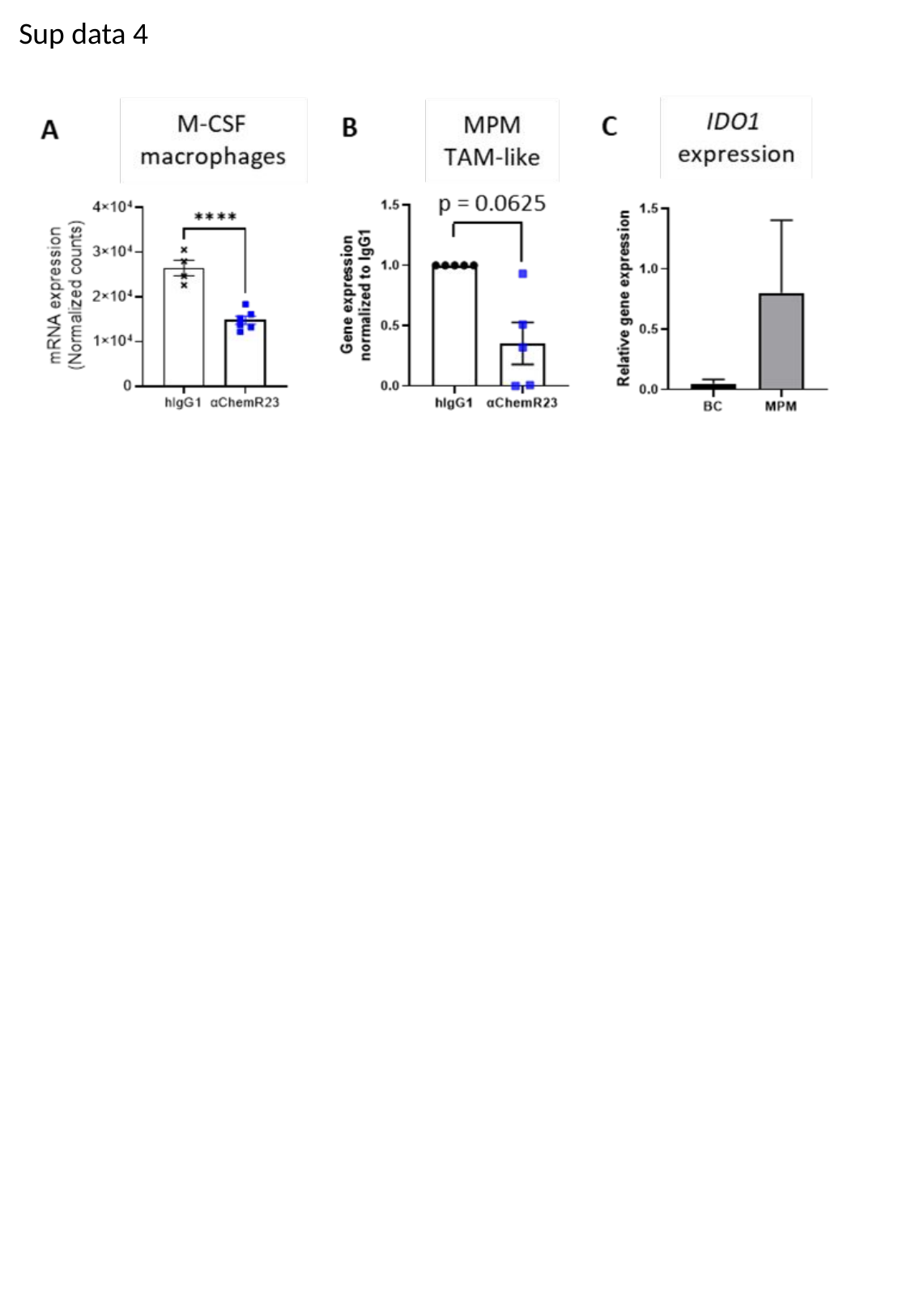

Sup data 4

## Slide 5
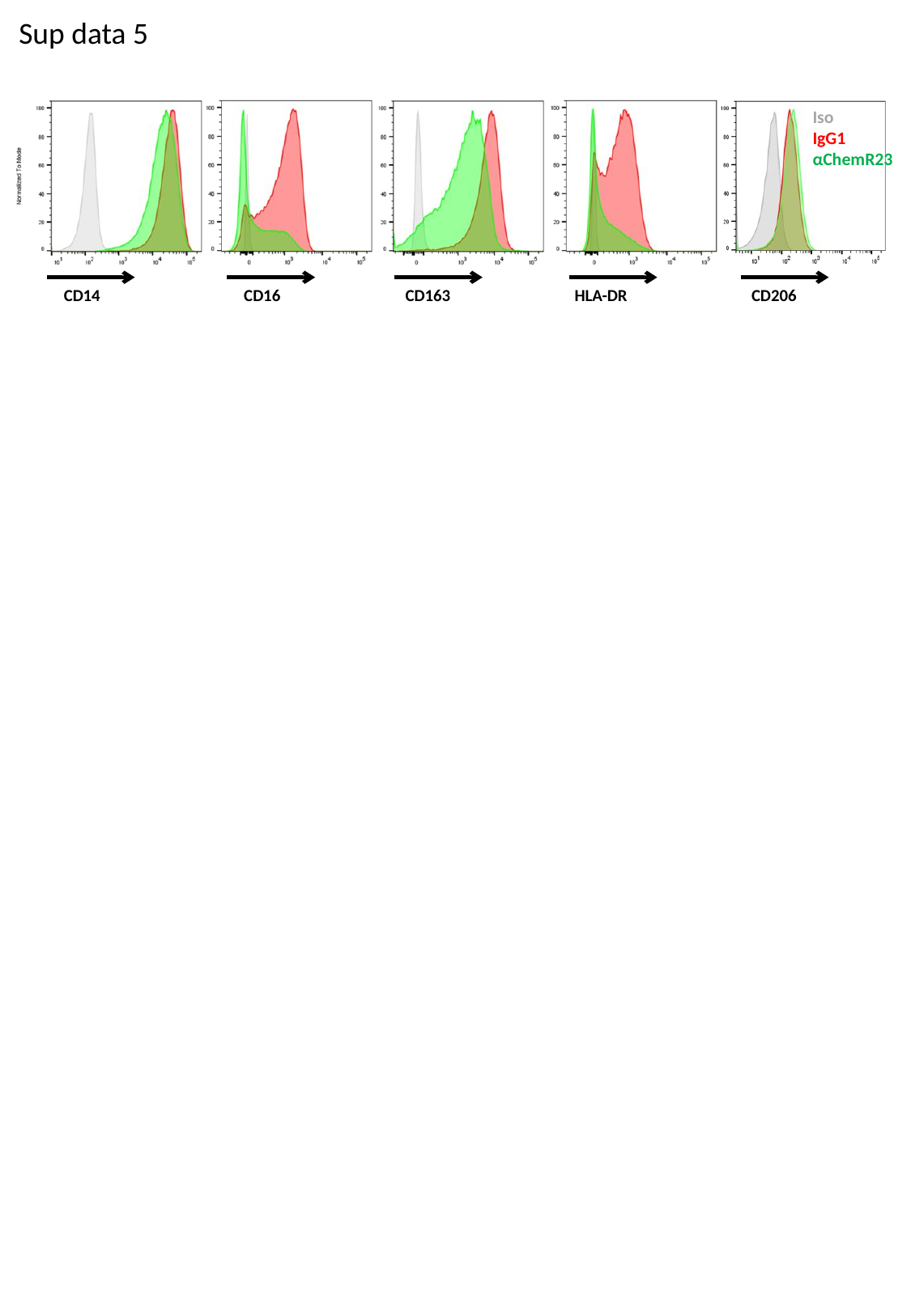

Sup data 5
Iso
IgG1
αChemR23
HLA-DR
CD14
CD16
CD163
CD206

## Slide 6
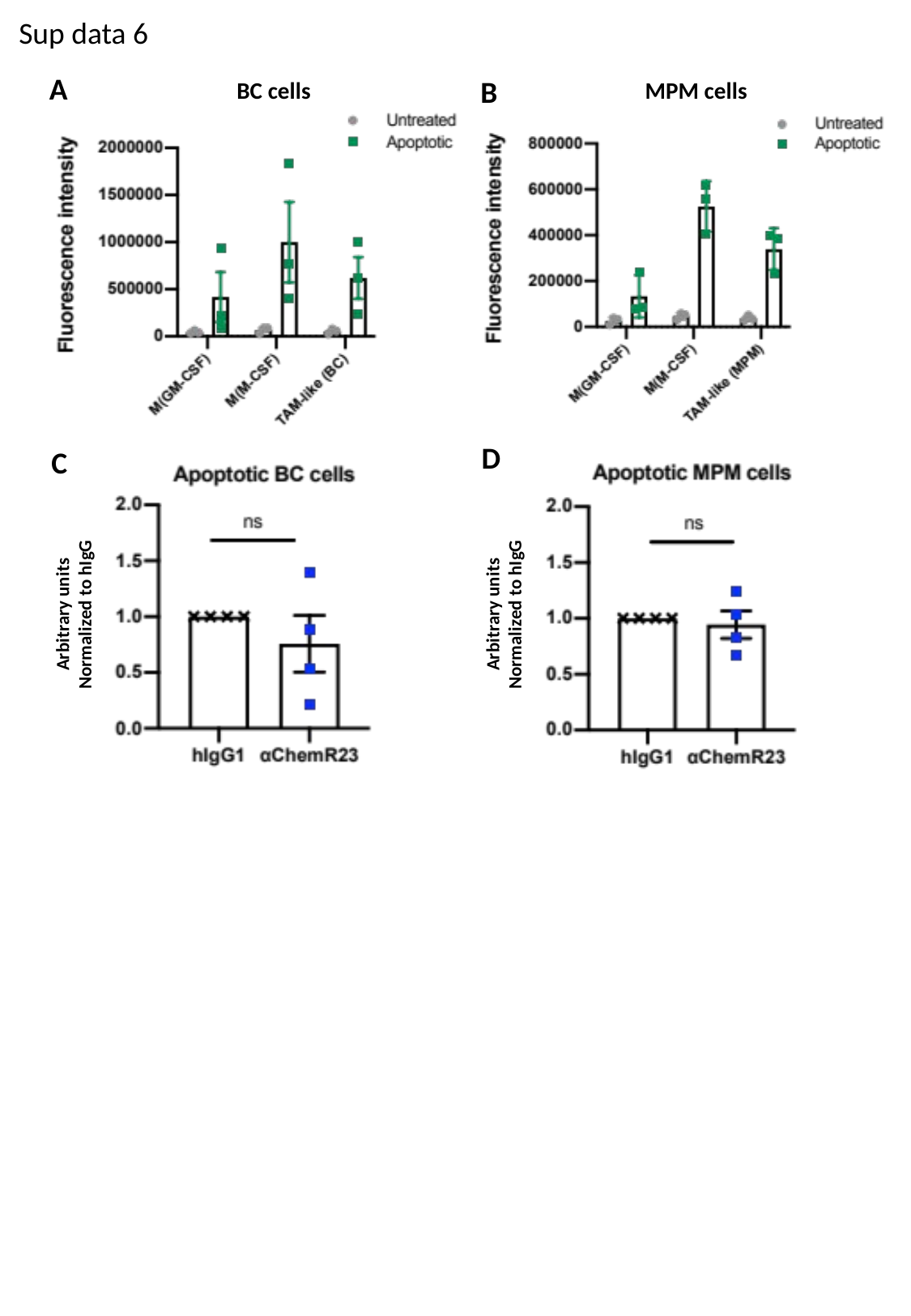

Sup data 6
A
B
BC cells
MPM cells
D
C
Arbitrary units
Normalized to hIgG
Arbitrary units
Normalized to hIgG

## Slide 7
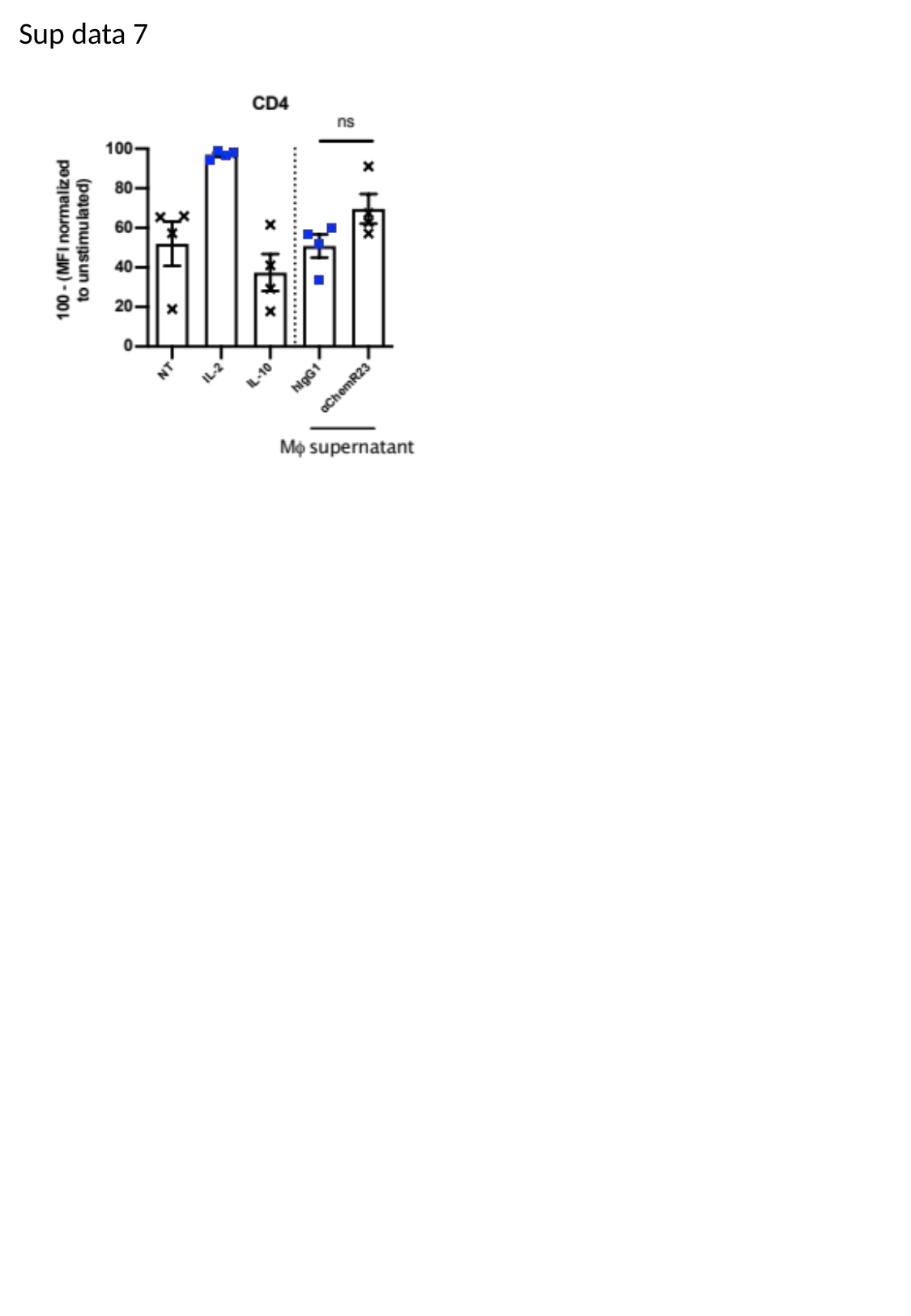

Sup data 7

## Slide 8
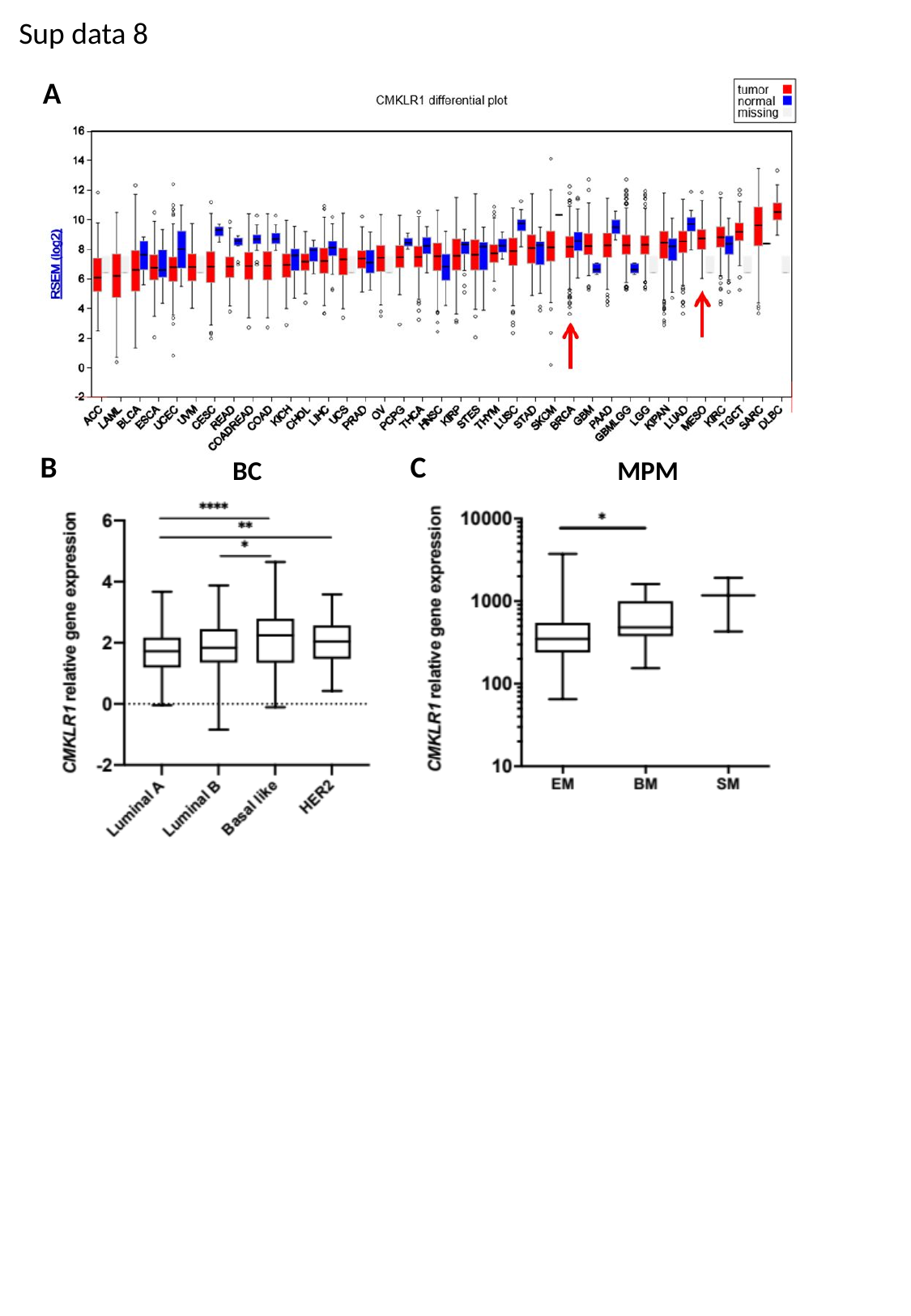

Sup data 8
A
B
C
BC
MPM

## Slide 9
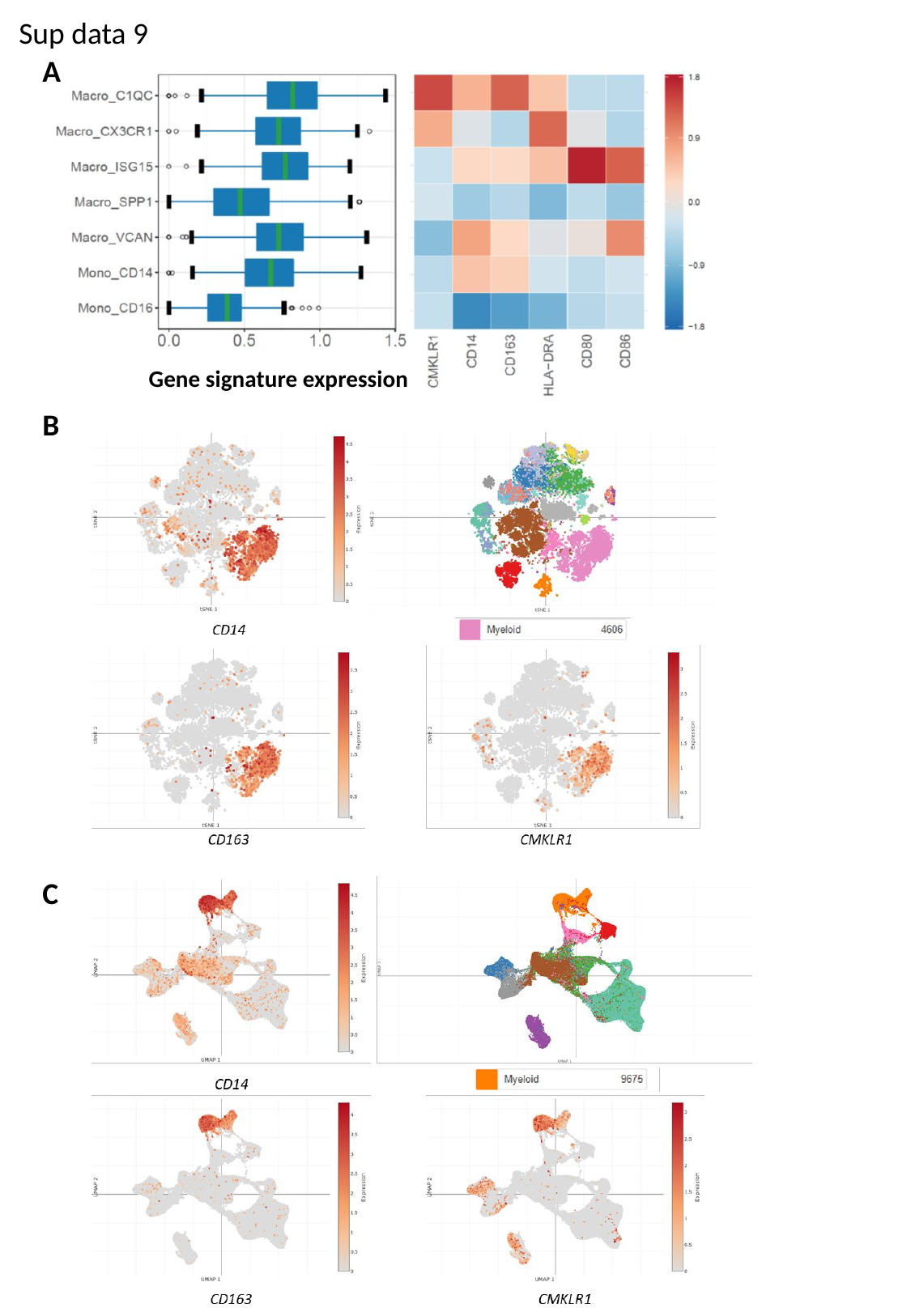

Sup data 9
A
Gene signature expression
B
C

## Slide 10
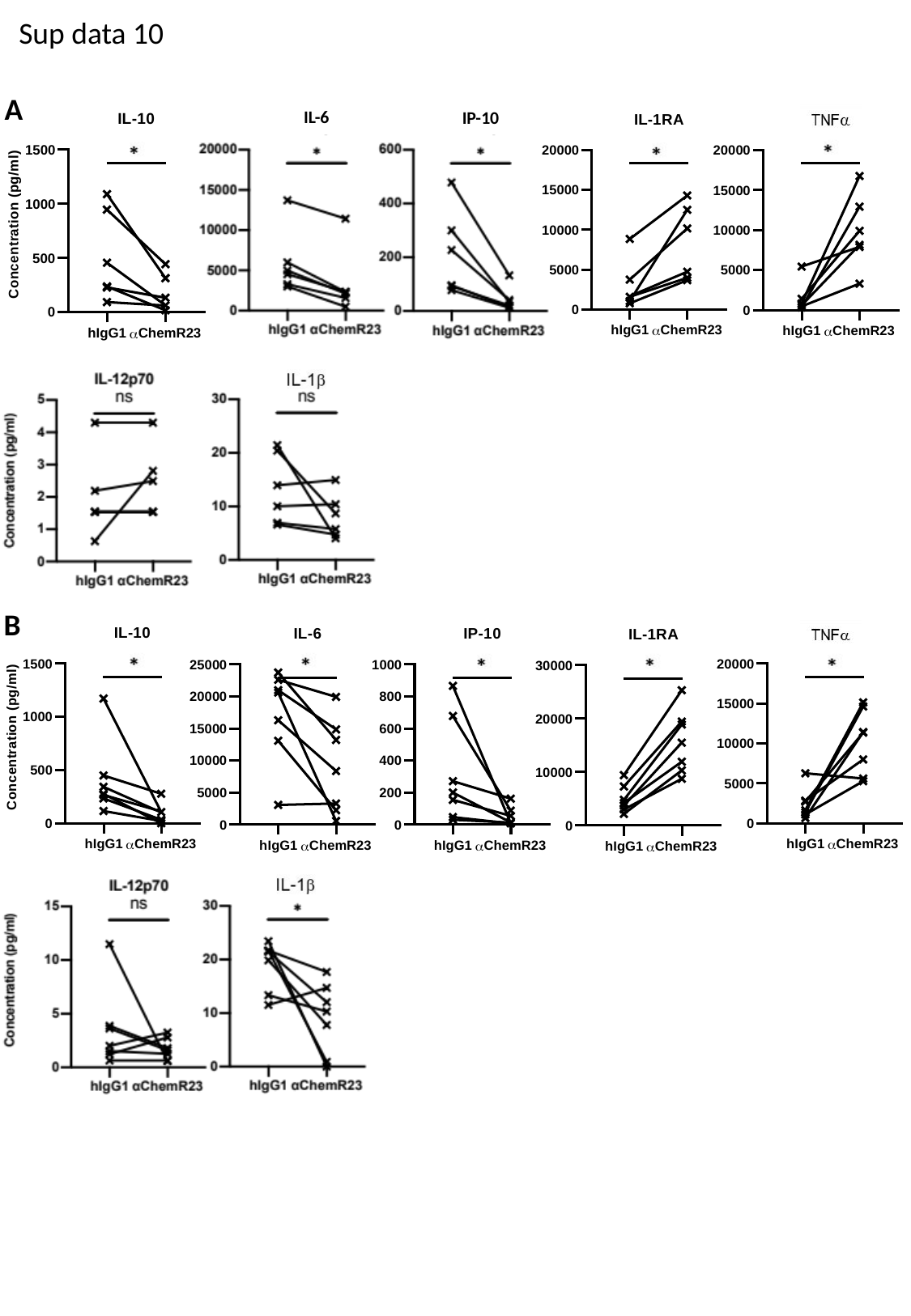

Sup data 10
A
IL-6
IP-10
B

## Slide 11
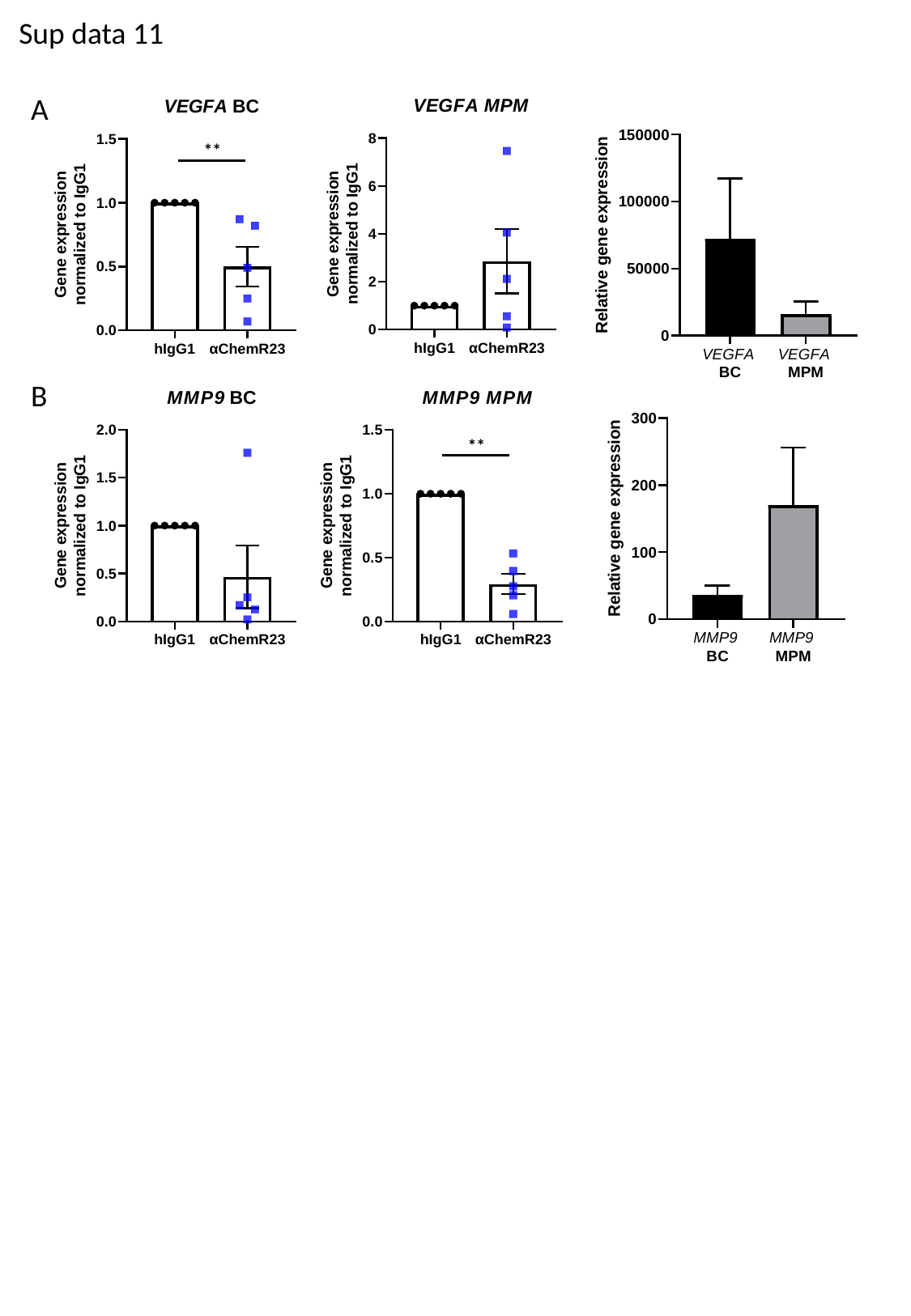

Sup data 11
A
**
B
**

## Slide 12
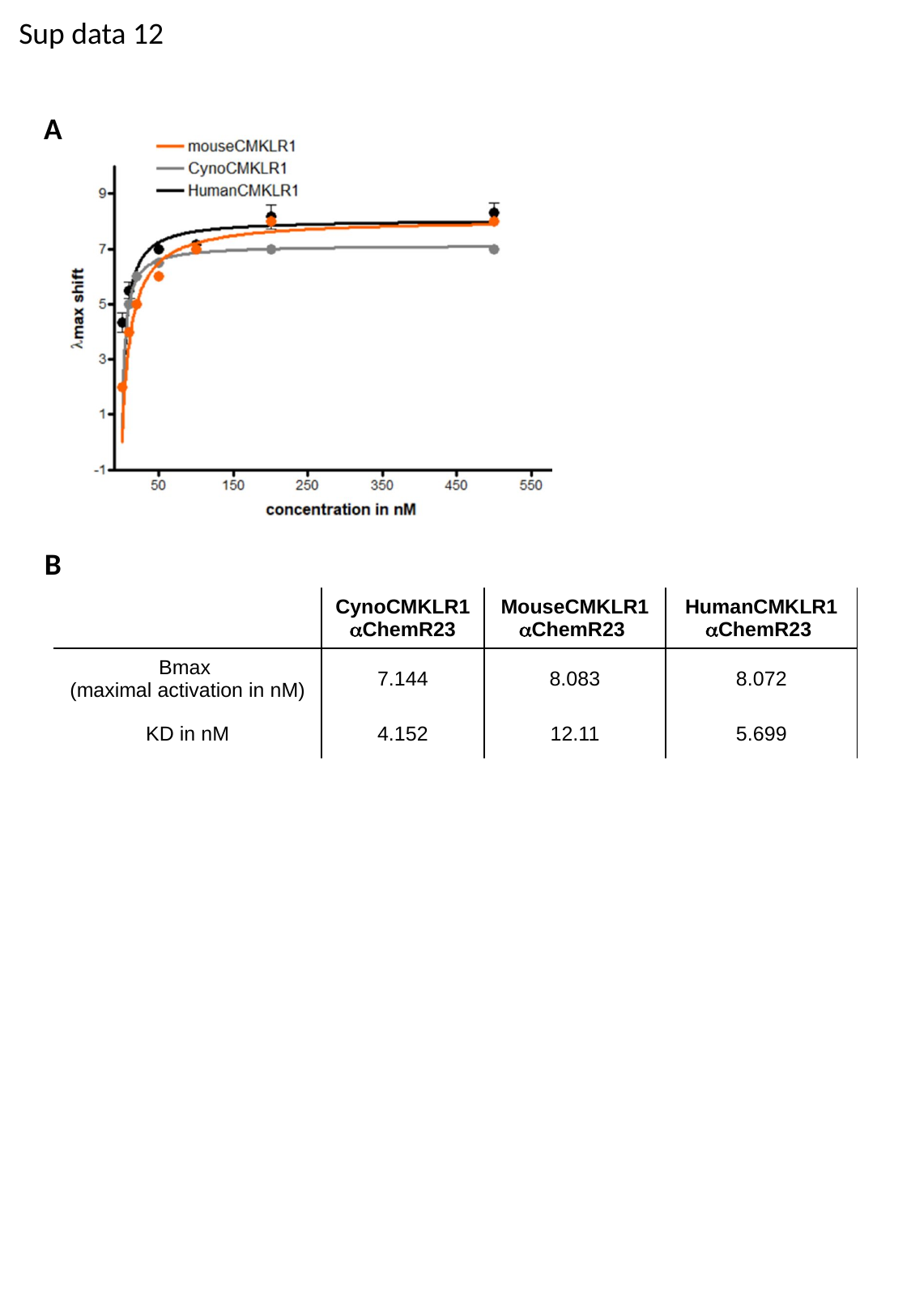

Sup data 12
A
B
| | CynoCMKLR1 aChemR23 | MouseCMKLR1 aChemR23 | HumanCMKLR1 aChemR23 |
| --- | --- | --- | --- |
| Bmax (maximal activation in nM) | 7.144 | 8.083 | 8.072 |
| KD in nM | 4.152 | 12.11 | 5.699 |

## Slide 13
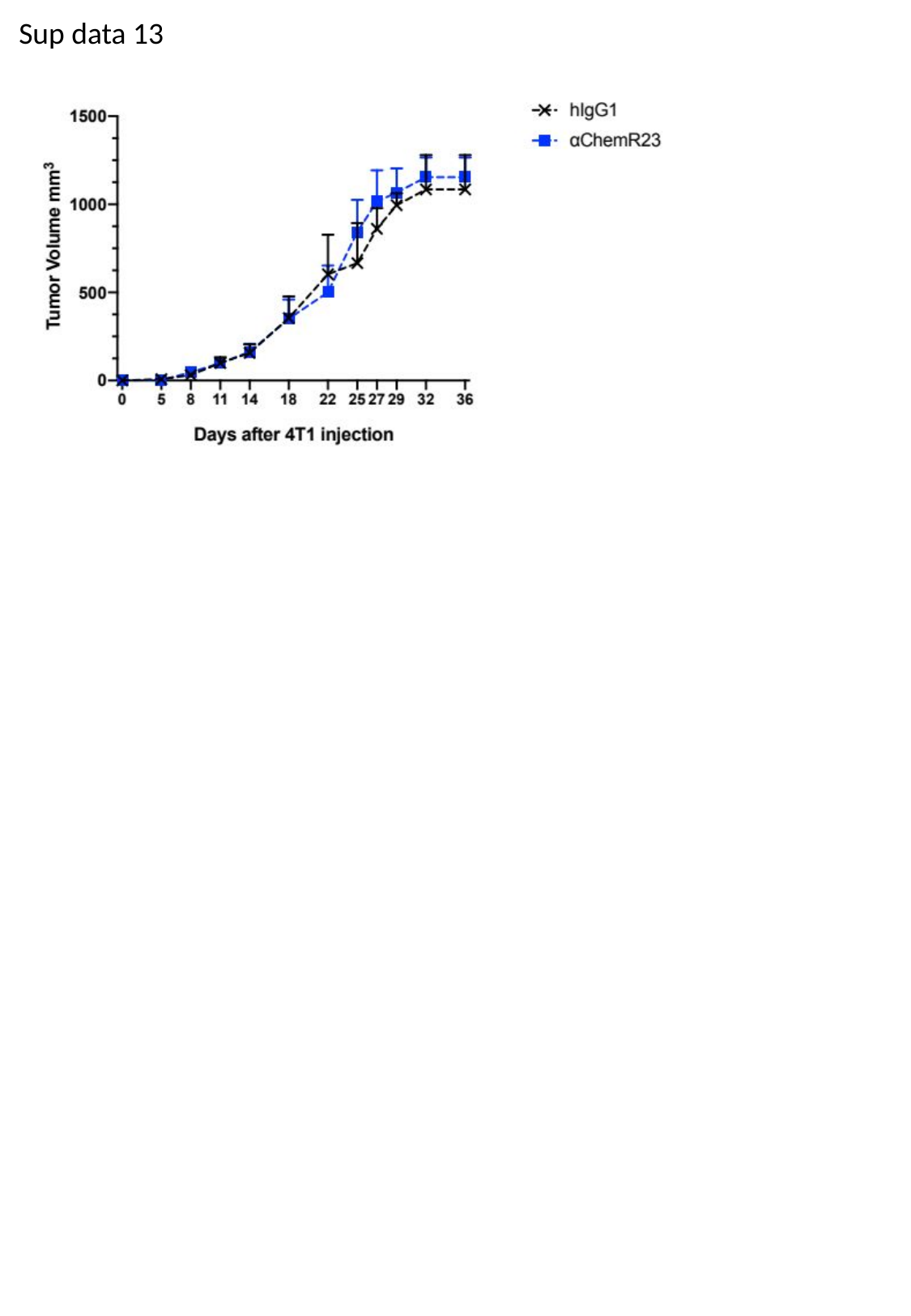

Sup data 13

## Slide 14
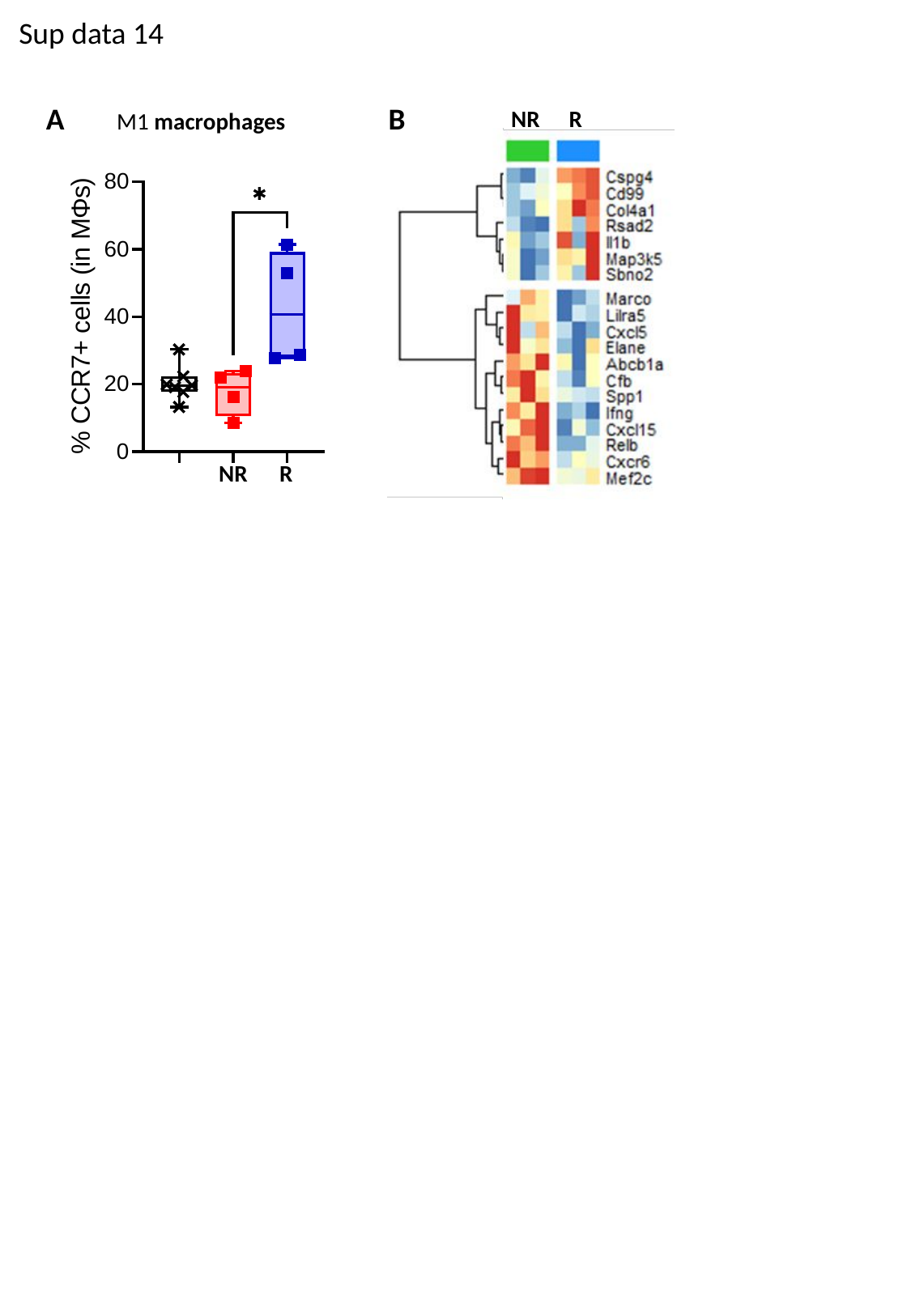

Sup data 14
A
B
R
NR
M1 macrophages
R
NR
